# Potential based, spatial simulation of dynamically nested particles

**DOI:** 10.1186/s12859-019-3092-y

**Published:** 2019-11-27

**Authors:** Till Köster, Philipp Henning, Adelinde M. Uhrmacher

**Affiliations:** Institute of Computer Science, University of Rostock, Albert-Einstein-Straße 22, Rostock, 18059 Germany

**Keywords:** Space, Simulation, Nesting, Force, Multi-level, Modeling, Attributed

## Abstract

**Background:**

To study cell biological phenomena which depend on diffusion, active transport processes, or the locations of species, modeling and simulation studies need to take space into account. To describe the system as a collection of discrete objects moving and interacting in continuous space, various particle-based reaction diffusion simulators for cell-biological system have been developed. So far the focus has been on particles as solid spheres or points. However, spatial dynamics might happen at different organizational levels, such as proteins, vesicles or cells with interrelated dynamics which requires spatial approaches that take this multi-levelness of cell biological systems into account.

**Results:**

Based on the perception of particles forming hollow spheres, ML-Force contributes to the family of particle-based simulation approaches: in addition to excluded volumes and forces, it also supports compartmental dynamics and relating dynamics between different organizational levels explicitly. Thereby, compartmental dynamics, e.g., particles entering and leaving other particles, and bimolecular reactions are modeled using pair-wise potentials (forces) and the Langevin equation. In addition, forces that act independently of other particles can be applied to direct the movement of particles. Attributes and the possibility to define arbitrary functions on particles, their attributes and content, to determine the results and kinetics of reactions add to the expressiveness of ML-Force. Its implementation comprises a rudimentary rule-based embedded domain-specific modeling language for specifying models and a simulator for executing models continuously. Applications inspired by cell biological models from literature, such as vesicle transport or yeast growth, show the value of the realized features. They facilitate capturing more complex spatial dynamics, such as the fission of compartments or the directed movement of particles, and enable the integration of non-spatial intra-compartmental dynamics as stochastic events.

**Conclusions:**

By handling all dynamics based on potentials (forces) and the Langevin equation, compartmental dynamics, such as dynamic nesting, fusion and fission of compartmental structures are handled continuously and are seamlessly integrated with traditional particle-based reaction-diffusion dynamics within the cell. Thereby, attributes and arbitrary functions allow to flexibly describe diverse spatial phenomena, and relate dynamics across organizational levels. Also they prove crucial in modeling intra-cellular or intra-compartmental dynamics in a non-spatial manner, and, thus, to abstract from spatial dynamics, on demand which increases the range of multi-compartmental processes that can be captured.

## Background

Space plays an important role in cell biological dynamics, such as cell signalling [1]. To study cell biological phenomena which depend on diffusion, active transport processes, or the locations of species, modeling and simulation studies need to take space into account. Over the last decade, a variety of spatial modeling and simulation methods and tools has been developed to support these simulation studies. They offer different approaches how to describe a spatial model, e.g., reaction-based, rule-based, or graphically, and they differ referring to what kind of spatial dynamics can be described, e.g., whether concentrations, populations, or individual particles are considered, whether a deterministic or stochastic approach is pursued, and whether movement takes place in discretized or continuous space [2, 3]. The kind of spatial dynamics that are supported by tools largely determines the results and questions that can be answered by a simulation study [4–6] and has been subject to different categorizations to structure the portfolio of spatial simulation methods from which the user can select.

TAKAHASHI et al. [7] distinguished spatial simulation approaches according to the representation of space. We adopt this structuring and slightly adapt it for the purpose of this paper as presented in Fig. 1. We distinguish between:

1. Stochastic non-spatial dynamics: To take the stochasticity of the modelled system into account, the propensity of reactions are sampled from exponential distributions and determine which reaction will occur at which time [9].

**Fig. 1:**
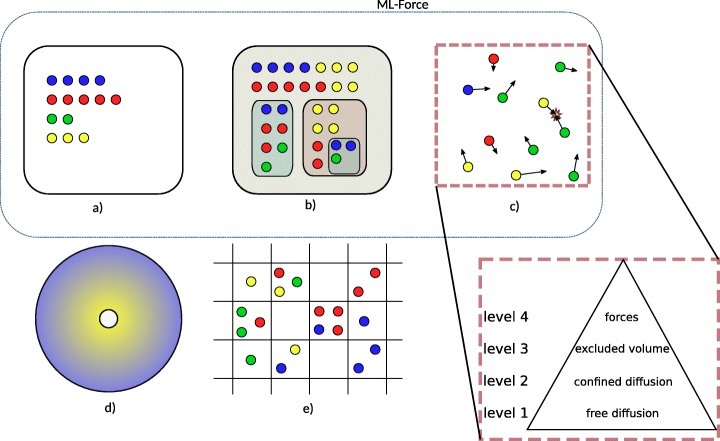
Representations of space which is based on and slightly adapts Figure 1 of [7]. We distinguish between (**a**) stochastic non spatial dynamics, (**b**) compartmental dynamics, (**c**) individual particles moving in continuous space, (**d**) partial differential equations, and (*e*) spatial stochastic dynamics. Type (*c*) individual particles moving in continuous space is further described by four levels, according to SCHöNEBERG et al. [8]. Each level builds upon the capabilities of the underlying ones, thus the representation as a pyramid. For example a method that provides excluded volumes should conceptually also be able to provide free diffusion and confined diffusion. ML-Force is characterized by a-b-c(4)
2. Compartmental dynamics: Many tools allow to constrain the dynamics of species to specific compartments. However also, compartments themselves can be subject to dynamics. For example, compartments can fuse and divide [10].
3. Individual particles moving in continuous space: Particles can be identified by their unique position in space, bimolecular reactions are triggered by collisions of particles, and typically particles diffuse by Brownian motion [11, 12].
4. Partial differential equations: Spatial gradients of concentrations are calculated deterministically [13].
5. Spatial stochastic dynamics: Multiple particles can occupy a position within a lattice space, and diffuse between neighboring positions [14].

Particle-based approaches (in the above categorization *c)*) are the subject of a further categorization suggested by SCHöNEBERG et al. [8] (Fig. 1, right lower corner). Spatial particle-based simulation tools are categorized into those that support

- *level 1 – free diffusion*: basic 3D diffusion of point particles and reactions between them are considered,
- *level 2 – confined diffusion*: diffusion can be constrained to compartments,
- *level 3 – excluded volume*: point particles are replaced by volumetric entities, and
- *level 4 – potentials for particle-particle interaction*: instead of a simple rejection of movements assuming rigid cells, potentials are used to determine the interaction of particles in terms of exclusion of movement or reactions.

According to this categorization [8], Smoldyn is characterised as *level 2* particle-based approach: point particles which do not hamper each others movement are equipped with binding and unbinding radii, they can be constrained to compartments. A more recent version includes collision strategies to reach *level 3* [15]. SpringSaLaD [16], ReaDDy [17], and SRSim [18] are based on forces to model the particle-particle interactions (*level 4*). Still, the levels introduced do not imply that a tool that supports *level 3* provides all interesting features that a tool working at *level 2* offers to study the system of interest. E.g., DONOVAN [19], whose spatial simulation works at *level 2*, supports arbitrarily complex 3D mesh geometries, whereas in ML-Space [20] (*level 3*) only rigid spheres are considered.

Merging both characterizations, approaches that combine different spatial representations can be characterized. The Two-Regime method [21] and KLANN et al. [22] allow to combine particle and RDME dynamics within one model: *c(3)-e*. Similarly in [23], particles and partial differential equations are combined to focus on the spatial region of interest: *c(3)-d*. [24] couples a partial differential equations solver and Smoldyn *c(2)-d*. ML-Space combines particles at *level 3* (as excluded volumes are considered), compartmental dynamics, and spatial, stochastic simulation (RDME): *b-c(3)-e* [20].

In addition, simulation environments offer the possibility to select different spatial semantics for one model specification. For example, in VCELL [13], rule-based models can be interpreted by a particle-based simulator (i.e., Smoldyn) or a partial differential equation solver, both confined to realistically geometrical compartmental structures (*c(2)*, *d*). This list is far from being complete, and the characterization of the simulation tools may only depict a specific state in their development.

**Fig. 2:**
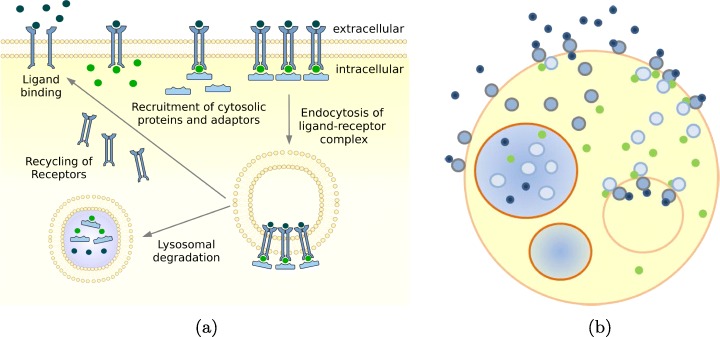
Illustration (combined from [27, 28]) of a receptor-ligand binding kinetics, which includes receptor protein coupling, internalization, and recycling, whose components can be interpreted as nested arrangements of objects within objects that interact at intersection points, can be approximated to spherical particles of various sizes, move in continuous space and undergo processes of creation, degradation, internalization, externalization, fusion, and fission. **a** Biological view, **b** Hollow spheres view
- *Rule-based approach with arbitrary attributes and functions*: As other spatial simulators, ML-Force supports rule-based modeling [29]. However in ML-Force, attributes are not constrained to a finite set of values. Similarly, as in ML-Rules [30], attributes are of arbitrary type and arbitrary functions are supported to determine the result and kinetics of reactions. The possibility to define attributed species and arbitrary functions increases significantly the expressiveness of a modeling formalism [31]. It allows to express spatial stochastic models (RDME) on a lattice (*e*) within a modeling approach that relies on a standard stochastic simulation algorithm for execution (*a*). By attributing species with indices that locate them in a specific point in the lattice (which represent a subvolume), e.g., *A*(*x*,*y*),*B*(*x*,*y*), reactions can be constrained to species within one subvolume, e.g., *A*(*x*_*a*_,*y*_*a*_)+*B*(*x*_*b*_,*y*_*b*_)→2 *B*(*x*_*b*_,*y*_*b*_)*@* if *x*_*a*_=*x*_*b*_∧*y*_*a*_=*y*_*b*_ then *k* else 0. Furthermore diffusion between neighboring subvolumes can be defined, e.g., *B*(*x*_*b*_,*y*_*b*_)→*B*(oneOf((*x*+1,*y*),(*h*−1,*y*),(*x*,*y*+1),(*x*,*y*−1)))*@k*_*d*_. Thus, attributed species and arbitrary functions can be used to add space to otherwise non-spatial approaches. This is an observation also made in colored Petri-Nets [32] and in expressive rule-based modeling languages such as ML-Rules [30].
  In ML-Force, equipping particles with attributes and arbitrary functions allows e.g. to describe some part of the cellular dynamics in a non-spatial manner. For example, the reaction *B*+*C*→*D* *@* *k* which takes place in particle A can be transformed into a non-spatial stochastic first order reaction (*a*). Therefore, the particle *A* is equipped with the corresponding attributes and a reaction is defined *A*(*b*,*c*)→*A*(*d*) *@* *b*·*c*·*k*. The function *b*·*c*·*k* calculates the propensity of the reaction to take place. The possibility to constrain reactions based on arbitrary functions defined on the attributes, allows us to include non-spatial stochastic dynamics (*a*) which brings ML-Force up to *a-b-c(4)* (see Fig. 1).

Against this background, we propose a new spatial particle-based modeling and simulation approach for cell biological systems, i.e., *ML-Force* (Fig 1). It combines:

- *Potential-based particle dynamics*: The excluded volumes that are represented by the large amount of macro-molecules within the cell affect the physico-chemical kinetics of various intracellular processes [25]. To capture the effects of molecular crowding, space exclusions need to be considered. In addition, interactions based on potentials are an effective means to study the formation of clusters [18]. Additionally, potentials allow us to capture directed movements, such as the transport of vesicles [26] (see “Results” section). Similar to ReaDDy [17], SRSim [18], or SpringSaLaD [16], ML-Force will use potentials for particle-particle interaction: *c(4)*.
- *Compartmental dynamics*: Intra-cellular space is further structured by compartments and vesicles, most of which are subject to frequent changes in terms of numbers, content, and inter-connectivity. A prominent example is the endosomal system. Vesicles form at the membrane. Here they acquire their cargo and engulf protein receptor complexes. Those are transported towards the inner cell, where part is degraded and part is recycled. The vesicles themselves move through stages of early sorting, recycling to late endosomes, closely interacting with each other – processes which include frequent fission and fusion [26] (Fig. 2). ML-Force uses the same force-based approach that it uses for the interaction of particles and global force functions, to model the compartmental dynamics. Similar to ML-Space, ML-Force supports both: compartmental and particle dynamics. However, unlike ML-Space it uses potentials *b-c(4)*.

## Methods

ML-Force adopts a rule-based approach to specify the spatial biochemical dynamics. Firstly the mathematical concepts and abstractions are presented. This is followed by a short description of its implementation and embedded domain-specific modeling language.

### Mathematical concepts and abstractions

As the name suggests, forces are central in ML-Force: they govern the interactions between and most of the activity of particles in a continuous manner. This is in contrast to the discrete event view chosen by simulators such as ML-Space or other agent-based approaches which interpret compartmental dynamics such as the proliferation of cells as discrete events [20, 33].

#### Representation of particles

ML-Force is a particle-based approach. Particles represent *hollow spheres*. As particles can contain other particles, they can be used to model cellular compartments. Particles are spherically symmetric and deformable, their size, properties and nested content may vary. Particles *p*_*m*_ are categorized into classes called *species*: *p*_*m*_∈*S*_*i*_. Species are characterized by a set of arbitrary attributes, *S*(*a*_1_,…*a*_*n*_), e.g., radius, phosphorylation sites, or phases. In our implementation of ML-Force we require particles to have at least the attributes radius *r* (non-zero) and density *ρ*. The information about its radius together with information about the system’s temperature and the viscosity of its environment allows to derive the diffusion coefficient of a particle. In combination with the density, this information is used to determine the actual velocity (see “Spatial propagation” section). Particles may contain other particles, *p*_*m*_(*v*_1_,…*v*_*n*_)[*p*_*j*_+…+*p*_*k*_]. All particles belonging to one class share the same attributes and attribute-types. Attribute values and content of particles may vary among particles of the same species. The interactions between and the activity of particles is governed by rules of behavior that operate on the particles, their attributes and their content. They rely on arbitrary functions for accessing and updating attributes and content of particles. Particles belonging to the same species share the same behavioral patterns.

#### Spatial propagation

For particles to interact they need to be in close proximity. In ML-Force, we base our model of propagation on the LANGEVIN equation [34]

1 $$ m_{i}\boldsymbol{\ddot{x}}_{\boldsymbol{i}}(t) = -\gamma {\boldsymbol{\dot{x}}}_{\boldsymbol{i}}(t) + \boldsymbol{f}(t) + \boldsymbol{F}_{\text{ext}}(\boldsymbol{x}_{i},t)\,{,}  $$

with the dot denoting time derivative and boldness indicating vectors. A Langevin equation is an ordinary differential equation with a random term added. This equation can be intuitively understood as an extension to NEWTON’s $\boldsymbol {F}(\boldsymbol {x},t)=m\boldsymbol {\ddot {x}}(t)$ by adding both friction (friction coefficient *γ*) which is linear to the velocity and a random force ***f*** which describes the accumulated effect of the system interaction with the individual particle. This interaction between the particles and the system results in the diffusion of the particles. In particular ***f*** is commonly sampled from a Gaussian distribution to model the aggregate system behavior.

Values for the constants involved can generally be found using known equations like STOKES-EINSTEIN relation ($ D_{i} =\frac {k_{B}T}{\gamma _{i}} =\frac {k_{B}T}{6\pi \eta r_{i}}$), as well as system properties like viscosity *η*.

The ***F***_ext_(***x***,*t*) expression is the sum of all external forces on a particle. Two types of external forces are distinguished:

- global forces are those, that act on one particle, independent of other particles. Global forces functions may be specified as arbitrary functions of the individual particle state (most importantly its position) and the global state of the simulator. This may be used to model, for example, some global current in a blood stream or some other directed cellular motion (as is done in “A model of vesicular transport” section below).
- pair-wise forces are those, that originate in the interaction of two particles like the non-reaction force (Eq. 2), reaction force (Eq. 7) or a division force (Eq. 6).

#### Non-reactive force

In order to model excluded volumes, we introduce a soft sphere potential between two non-reacting particles. This is a common and natural way to model excluded volumes in force-based approaches. In addition, a hard-sphere potential would imply more efforts numerically, as it would lead to a discontinuous force. An established choice for the modeling of elastic (soft-sphere) collision is the HERTZ collision theory [35]:

2 $$  F_{non-react}(d)=\frac{4}{3}E^{*}\sqrt{r\cdot d^{3}}  $$

where *d* is the overlap of the particles, $E^{*} = \left (\frac {1-\nu _{1}^{2}}{E_{1}}+\frac {1-\nu _{2}^{2}}{E_{2}}\right)^{-1}$ results from the YOUNG’S modulus *E* and POISSON’S ratio *ν* and $r =\left (\frac {1}{r_{1}}+\frac {1}{r_{2}}\right)^{-1}$ from their radii. However, for many sparse applications, any kind of polynomial would suffice, as the exact dynamics of the elastic collision do not matter, when the mean time between collisions is sufficiently large.

### Reactions in mL-Force

Reactions between particles in ML-Force include zero, first and second order reactions. ML-Force supports a rule-based description of models. Whereas lower order reactions are sampled from an exponential distribution, second order reactions are calculated, similarly as the movement of particles, based on forces.

#### Lower order reactions

Simulation of zeroth and first order reaction, e.g., ⊥→*p*_*j*_, or *p*_*i*_→*pi*′, or *p*_*i*_→*p*_*j*_+*p*_*k*_ is a well understood problem (e.g., in SpringSaLaT [16]). The most basic approach is to sample the rate every (sufficiently small) time-step and check it against a random number.

In ML-Force, rates can be defined by arbitrary functions that access the attributes and contents of the particles involved in a reaction, e.g., for a first order reaction:

3 $$ {{}{\begin{aligned} p_{i}(v_{1},\ldots v_{n})[p_{1} + \ldots + p_{m}] \rightarrow \ldots @ \lambda (k, v_{1},\ldots v_{n},[p_{1},\ldots,p_{m}]) \enspace. \end{aligned}}}  $$

here *λ* denotes a function defined by the user, *k* a parameter, which might refer to the system globally, e.g., in terms of temperature and viscosity, or to a kinetic constant. The result of the function *λ* serves as the parameter for the exponential random distribution, that describes the probability density of the time until the reaction occurs. Thus in ML-Force, zero and first order reactions are treated semantically the same as in stochastic simulation algorithms [9]. In stochastic, non-spatial and spatial, simulation approaches (see Fig. 1a and e) exponentially distributed stochastic events form the basis of all, including second order, reactions.

In ML-Force the rate is sampled every time step. Instead of propagating the simulation until the exact time point a reaction occurs, we check (stochastically), if the reaction occurs in the current time step. Sampling the rate every time step is useful if the information (*v*_1_,…,*v*_*n*_,[*p*_1_,…,*p*_*m*_]), on which the rates depend, frequently changes. If this information remains largely constant over time, a scheduling approach may be more efficient computationally, as events do not have to be rescheduled frequently [36]. In executing ML-Force models so far, zeroth and first order reactions have only been responsible for a small fraction of the required computing-time.

In addition to computational considerations, the error introduced by this procedure needs to be considered. Due to the discretization of time (explicit time steps) no-more than one reaction per particle may take place per time step. This is important to keep in mind for future models. In the application domain the mean time between reactions tend to be large compared to the very small time steps that result from the fast diffusion of particles. As long as this is the case, the error is negligible. With resonable technical effort, this problem could also be avoided by allowing multiple reactions per time step.

One kind of lower order reaction is the changing of particle size, in terms of a particle’s radius *r*. In ML-Force arbitrary functions can be applied. The new value of the radius *r*, i.e., $v^{\prime }_{1}$, of a particle *p*_*i*_∈*S*_*j*_(*r*,…) is calculated in dependence of some global parameters *k*, and the current state of the particle in terms of its attribute values *v*_1_,…*v*_*n*_, and its content *p*_1_…*p*_*m*_, such that

$$ {{\begin{aligned} &p_{i}(v_{1},\ldots v_{n})[p_{1} + \ldots + p_{m}] \\ &\rightarrow p_{i}(\lambda (k,v_{1},\ldots v_{n},[p_{1},\ldots,p_{m}]), v_{2} \ldots,v_{n}) [p_{1} + \ldots + p_{m}] \ldots @ \lambda (\ldots) \enspace. \end{aligned}}}  $$

By default, whenever a particle is created, it is first put into the system as a very small particle and then quickly grows continuously to its desired size. This continuous change of particle radii is also used, whenever the radius of a particle is changed in the model by any kind of reaction. Carrying the changes out suddenly (so not slowly over multiple integration steps) would potentially lead to integration errors, especially in systems with nesting. A sudden change in size could for example lead to a high particle overlap, which in turn would create a high unrealistic potential energy leading to a strong force and thus a disturbance in the system, e.g., numerical heating. Slow changes in size, allow for each artificial change in energy to slowly dissipate e.g., in friction due to the numerically very stable Langevin model. Naturally, it is also possible to create a particle within or at the surface of another particle. Those new positions are chosen randomly. At the surface this means that the new particle’s shell barely touches the old particle’s shell.

#### Compartment division

An interesting kind of first order reaction is that of a cellular compartment (or specifically a cell) dividing.

4 $$ {{}{\begin{aligned} p_{i}(v_{1},\ldots v_{n})[p_{1} + \ldots + p_{m}] \rightarrow p_{i1}\left(\lambda_{11} (y), \ldots,\lambda_{n1} (y)\right)\left[\lambda_{c1} (y)\right] \\ + p_{i2}\left(\lambda_{12} (y), \ldots, \lambda_{n2} (y)\right)\left[\lambda_{c2} (y)\right] \qquad @ \lambda (\ldots), \{ a \} \, {,} \end{aligned}}}  $$

with *y*=*k*,*v*_1_,…*v*_*n*_,[*p*_1_,…,*p*_*m*_] or in shorthand, omitting attributes and contained particles

5 $$  p_{i} \to p_{i1}+p_{i2} @ \lambda (\ldots), \{ a \} \enspace.  $$

In ML-Force this is realized in a continuous and nesting compatible manner. As a first order reaction, the event that the division starts is sampled from an exponential distribution. The simulator temporarily introduces an “ignoring” relation between particles. This can be best explained using an example: Imagine a particle *p*_*i*_ containing many particles $\hat {p}_{1} \ldots \hat {p}_{m}$. If now the reaction for *p*_*i*_ to split into *p*_*i*1_ and *p*_*i*2_ triggers, a few things happen. First of all, each particle $\hat {p}_{1} \ldots \hat {p}_{m}$ gets assigned to remain in either *p*_*i*1_ or *p*_*i*2_. By default, this assignment happens randomly. If required by the model, a specific splitting function may be specified by the user. Therefore, two arbitrary functions, i.e., *λ*_*c*1_,*λ*_*c*2_ are applied. The results of these applications form the new content of the newly generated particles, i.e., *p*_*i*1_ and *p*_*i*2_ respectively. The functions *λ*_*i*1_ to *λ*_*n*1_, respectively *λ*_*i*2_ to *λ*_*n*2_ determine the new attribute values of the particles. If attribute values remain the same, simply the old values *v*_*i*_ can be used.

Each $\hat {p}_{i}$ that will remain in *p*_*i*1_ is set to ignore the particle *p*_*i*2_ and vice versa. Also *p*_*i*1_ and *p*_*i*2_ are set to ignore one another. Now an external force is applied to push *p*_*i*1_ and *p*_*i*2_ apart

6 $$  F_{\text{divison}} = m_{i}\cdot a_{\text{division}} \enspace.  $$

The force is calculated based on the acceleration *a* that is part of this particular reaction type (Eq.: 4, 5) in ML-Force and the mass of the involved particles (which again depend on the respective radii and their density *ρ*). This is chosen, instead of a uniform force, to create a uniform motion of all particles. In principle however, any force expression suitable to the model could be used here, like a half sine wave for example. Once *p*_*i*1_ and *p*_*i*2_ are fully outside one another, all the newly introduced ignore relations are removed and regular propagation proceeds.

The principle of continuous change in particle size also applies here. At the beginning both particles have the same size. If *p*_*i*1_ and *p*_*i*2_ are to change their size throughout the division (see example in “The yeast model” section), this change is carried out throughout the division process. Initially, both *p*_*i*1_ and *p*_*i*2_ share position and radius. The further they move out, the closer their radii become to the desired ones. The fusion of compartments could be added in an identical (if reversed) fashion. However so far, it has not been implemented yet.

#### Bimolecular reactions

Handling bimolecular reactions in particle-based approaches is rather challenging. Whilst solutions exist when not considering excluded volumes (e.g. Smoldyn [12]), there has not yet been agreement among the community on the best way to approach this problem. This is evident from the many different approaches of recent tools [5, 12, 16, 17, 37]. Two processes contribute to the macroscopic rate,

- the *diffusion process* describes how likely it is that two particle actually move into the vicinity of each other. This upper bound to the reaction rate is well described by the Smoluchowski-Theory [38] (elaborated in the Additional file 1)
- the *microscopic activation process* describes how likely it is for two particle to react once they are close to each other [39, 40].

The rate is considered to be diffusion limited if every interaction results in a reaction, all other cases are considered to be activation limited. We propose to use forces for both processes. This means also the microscopic activation process is based on forces. In case a reaction can occur between two particles, the non-reactive force (Eq.: 2) has to be replaced by a reactive force. We used a rather simple approach which is calculated based on a degree of overlapping required *d*^∗^ and the work to cross the energy barrier between the two particles. Both, the degree of overlapping and the energy barrier, are specific for the reaction to occur and have to be defined by the modeler.

7 $$  F_{react}=\frac{W}{d^{*}}  $$

where *d*^∗^ denotes the required overlap of the particles and *W* the work required to cross the energy barrier. It should be noted due to the modular design (see “Simulator and implementation” section) within the simulator these functions can easily be changed. Suppose we were to look at the reaction *p*_*i*_+*p*_*j*_→*p*_*k*_{*d*^∗^,*W*}. Whenever *p*_*i*_ and *p*_*j*_ overlap, they start to repel each other with the force *F*_*react*_. Once they overlap more than *d*^∗^, we consider the reaction to be triggered. Thus, the energy barrier and the required overlap of the microscopic interaction potential determines the likelihood of the reaction to take place. The higher the energy barrier, the less likely the particles will overlap sufficiently. If the energy barrier *W* is negligible, the diffusion is the only limit to the macroscopic reaction rate in the reaction process, it is *diffusion limited*.

This type of bimolecular reaction handling with forces, allows also for self-consistent handling of dynamic nesting (and “unnesting”) reactions, i.e., one particle shuttles into (or out of) another particle. In principle the same concepts apply as for the regular bimolecular reaction. Looking at the reaction *p*_*i*_+*p*_*j*_→*p*_*i*_[*p*_*j*_]{*d*^∗^,*W*} (and the same for *p*_*i*_[*p*_*j*_]→*p*_*i*_+*p*_*j*_{*d*^∗^,*W*}), first the particles need to diffuse into proximity of one another. Once particles overlap, they repel each other. If *p*_*i*_’s diameter has fully passed over *p*_*j*_’s outline, the nesting reaction is triggered. However at this point *p*_*j*_ is already fully contained in *p*_*i*_ so there is no jerkiness in the simulation. For example, if there is no unnesting reaction defined for these particles, the only thing, that changes, is that, if *p*_*j*_ were to move towards again touching *p*_*i*_, it would perceive the force *F*_*non*−*react*_ (pulling inwards). This ensures a soft-sphere-style excluded volume. Similar as for the regular *p*_*i*_+*p*_*j*_→*p*_*k*_ type bimolecular reaction, a high energy barrier makes for a less, and a low for a more likely reaction.

The advantage of this force-based approach, is the smooth integration of the nesting process. On the other hand, it does require a very small timestep, compared to other methods. Furthermore, in the current situation, the parameterization of bimolecular reactions is not optimal. Ideally, the modeler would specify the macroscopic rate, instead of the microscopic barrier. In the future we plan on investigating this further and, using principles from thermodynamics, provide an automatic conversion method. This would mean, by modeling the system as a thermodynamic ensemble we could determine the statistical likelihood of a particle surpassing a certain energy threshold during interaction and put this into explicit mathematical relation to the reaction rate.

### Simulator and implementation

The simulator has been implemented using C++14 and has been tested on both Windows and Linux (using CMake-build system) and is available at https://git.informatik.uni-rostock.de/mosi/ml-force-publication.

The user-interface is built around a rudimentary embedded domain specific language (DSL). An example and explanation can be found in the provided code snippet below. In the long run, it would be advantageous to create an external DSL using techniques like transpiling, to still have the performance benefits of a compiling language. A more in-depth comparison and discussion of DSLs can be found in the literature [41]. The developed embedded DSL bears some similarities with the *ℓ*-language [42] which also supports an imperative rather than a declarative approach towards modeling.

**Fig. 3:**
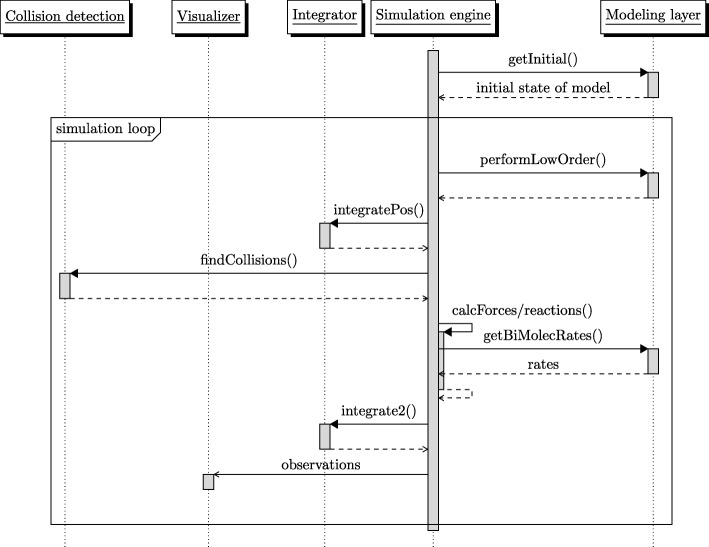
The different components of the software and their respective tasks, as called upon by the main simulator in an UML sequence diagram. The different components of the software are interchangeable and only interact via their interface to the main *Simulator*-component. This simulator also provides the layer between the model (as specified using the DSL).The software implementation is described on in “Simulator and implementation” section. The second integration of the particles is optional. For example the current integrator advances the velocity in the second integration step

As can be seen in Fig. 3, the software pursues a component-based design with a clear separation of concern [43]. Its components are arranged around the main *simulation engine*. The simulation engine itself is very basic and designed to make as little assumptions about the underlying semantics as possible. This simulation engine interacts with the components via a well defined, small interface. Each of the key ingredients of the simulation is separated into exchangeable components, namely the integrator, collision detection engine, and the visualizer, as well as the overarching modeling layer which provides the interface between the simulation engine and DSL. They are invoked by the simulator and implement the functionality introduced in the previous section. The simulator is responsible for executing the lower and higher order reactions by calling the specified functions of the model. First it is checked whether lower order reactions trigger in this step. If so, those are executed. Based on the forces a numerical integrator calculates the new positions of the particles. To identify the particles that interact, a collision detection engines is invoked. Afterward the simulator calculates the bimolecular forces and executes the biomolecular reactions accordingly. The calculated state can be subject to visualizations or other reporting mechanisms.

**Integrator** Currently integration is carried out using a two-step kick-drift-kick integrator [44] of the stochastic LANGEVIN equation. This integration includes velocity, and is thus fairly costly numerically. However as an alternative, one could select an integrator with immediate dissipation of momentum, that would allow for a considerably larger timestep. The choice for the more complex integrator was made as ML-Force makes heavy use of various forces and carrying out the more complex integration, helps in terms of correctness. In the current implementation the time step is chosen adaptively based on the smallest particle and the fastest velocity currently in the system, as $\Delta t = \frac {r_{\text {min}}}{v_{\text {max}}\,g}$ with *g* as the parameter of *granularity*, that describes the precision of the integration.

**Collision detection engine** Finding possible interactions of particles using the collision detection engine is one of the key drivers of runtime in the current implementation. Several collision detection engines have been implemented, using different algorithms and parallelization. For example, for highly nested particles, the collision-detection problem lends itself to be solved by a specifically tailored algorithm and a massively parallel execution on the GPU [28].

**Visualizer** Currently 3 basic visualizers have been created. One to just create an output text file, and two based on CImg [45], one for live viewing and one for generating movie files. The visualizers are encapsulated in a separate thread to minimize overhead. One more advanced visualization has been implemented that maps the simulation to 3D space using OpenGL [46]. It offers some useful features for the case studies (see “Results” section), such as tracking individual particles in space.

**Modeling layer** The modeling layer makes heavy use of *λ*-functions in order to encapsulate model specific behavior into single function calls for the simulator. *λ*-functions here are a C++11 feature that allows to define anonymous functions, that can be used as parameters in a functional-like programming style. This is facilitated by various templates that have been defined in the DSL. For example, after each reaction, the simulator invokes the *post reaction function* (see e.g. afterFuncA in listing 2). In the modeling layer this function is specified by the user and denotes the results of applying a rule, such as particles changing state, being degraded or created.

This simplifies the implementation of the simulator and gives a unified and lean interface for implementing further features. The simulator also provides a set of units (via the units:: namespace) to the modeler, allowing for consistent parameterization. For simplicity the reactants in the DSL are always denoted as *A* and *B*. The particular type of *A* and *B* is then specified via the *as_A* etc. functions. In general the DSL is very expressive, as all rates and reactions are described as *λ*-functions. These can be of arbitrary complexity. However, it should be noted that the current DSL presents only a proof of concept rather than a language that allows a succinct description of ML-Force models. Therefore, further efforts will be dedicated to improving the design of the language. 
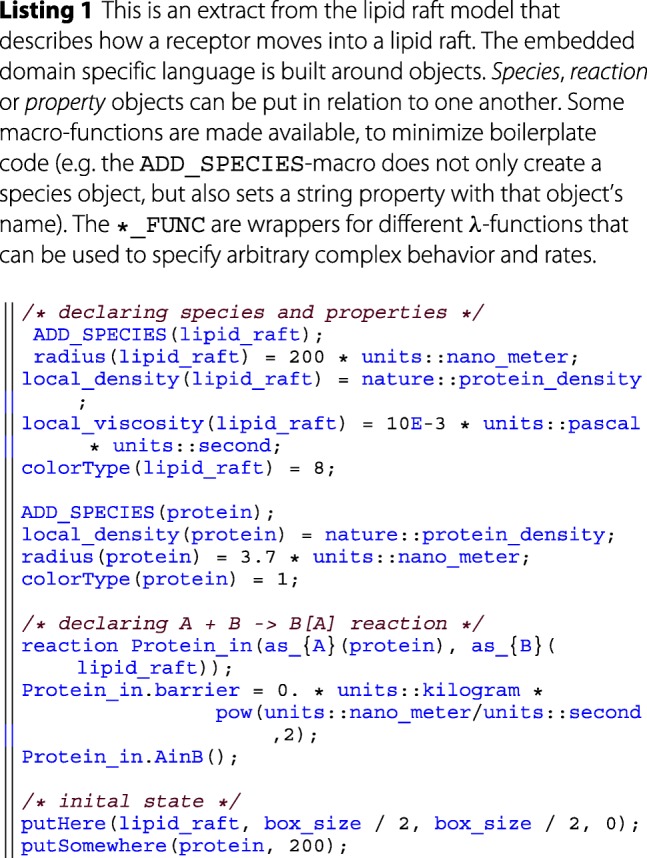


## Results

In order to test the ML-Force simulator and its implementation, we conduct a few case studies. These include simple test cases to investigate correctness and more complex models. The latter are inspired by realistic cell biological models, make use of the features in ML-Force, and, thus, demonstrate their usefulness. Both, the basic test models and the more realistic ones, can be found in the Additional file 1.

### Testing correctness

As other particle-based simulation approaches [16, 17, 37, 47] we test the correctness of our simulator, based on a set of simple reactions which we simulate to compare the achieved results to theory. To those tests belong the creation of a particle with a constant rate as a 0 order reaction ($\emptyset \xrightarrow {\text {k}} A$), the decay with a rate $A \xrightarrow {\text {k}} \emptyset $ as first order reaction, and irreversible and reversible second order reactions *A*+*B*⇔*C* in the diffusion limited (intrinsic rate *k*_*int*_=*∞*) case. A comparison of simulation results and theory shows overall a good agreement with the theory (see Additional file 1). However, those tests also reveal the decisive role of selected parameters to determine forces (“Bimolecular reactions” section) and time steps. For their selection, suitable computational support should be provided. As a form of pre-processing step, energy barriers respectively accelerations for individual reactions could be generated by automatically fitting simulation results to theory. A similar strategy can help selecting suitable time steps, so that the diffusion of particles is correctly simulated (see Additional file 1).

### A model of vesicular transport

HEINRICH and RAPOPORT proposed a model of vesicular transport [48]. Although the model describes a spatial process, it was formalized by means of differential equations.

The vesicular transport model refers to two cellular compartments which exchange membrane-bound soluble N-ethyl-maleimide–sensitive factor attachment protein receptors (SNAREs) and cargo proteins with the help of differently coated vesicles. These are budding from the compartments and move, driven by motor-proteins, to the other compartment where they are fused. Thereby the coat of vesicles defines, which type of SNAREs (X or Y), cargo proteins and motor-proteins are bound to them. Since different types of motor-proteins move in different direction, SNARE X and Y accumulate in different compartments.

By modeling this process in a spatial regime, the problem starts, as in each particle-based approach, with the description of the species. Each species needs a radius and a diffusion coefficient. The ML-Force simulator determines the diffusion based on the temperature, solvent viscosity and particle radius according to the STOKES-EINSTEIN equation (see Additional file 1). The cell, compartments and the vesicles are represented as particles while the SNAREs are attributes of particles, which change during the budding and fusion processes. As KLANN et al. [2] has already shown through the translation of the original ODE model into a spatial agent-based model, this transport relies on a directed movement of vesicles. Therefore Klann et al. added a cytoskeleton structure to the cell on which the motor-proteins moved along. In ML-Force the direction is determined by forces which take the type of particles and their attributes into account. An external force field is introduced which is shaped like a dipole field with the compartments as poles. Based on the coat of the vesicles a force along this field is added to their movement, which lead them to one of the compartments. To test the sorting mechanism of this simple vesicular transport model we study a system with 2 types of SNAREs (X and Y) with the same amount. As in [48], we initialize the model: the first compartment is nine times bigger (*V*_1_≈ 0.118 *μm*^3^) than the second one (*V*_2_≈ 0.013 *μm*^3^) and contains 90% of the SNAREs (X and Y) ($N_{1}^{X/Y}$ = 90000 and $N_{2}^{X/Y}$ = 10000). Since the budding process of the vesicles depends on the volume of the compartments, they should reach an equal size and the SNAREs should be sorted.

**Fig. 4:**
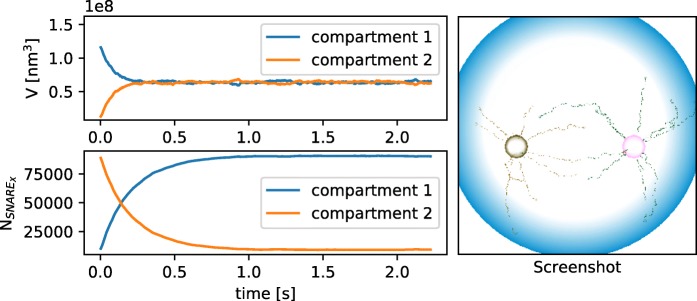
Volume and amount of SNARE _*X*_ over time and Snapshot from the vesicle transport model. In the case of a directed movement the volume of the compartments become equal and SNARE _*X*_ accumulate in compartment 1 as shown on the left. The right side shows a screenshot from the simulation where you can see the movement along the external force field. The blue sphere is the cell with a radius of 2.5 *μm*

As shown in Fig. 4 both compartments reach an equal volume and SNARE X accumulates in compartment 1 while SNARE Y accumulates in compartment 2 (not shown).

**Fig. 5:**
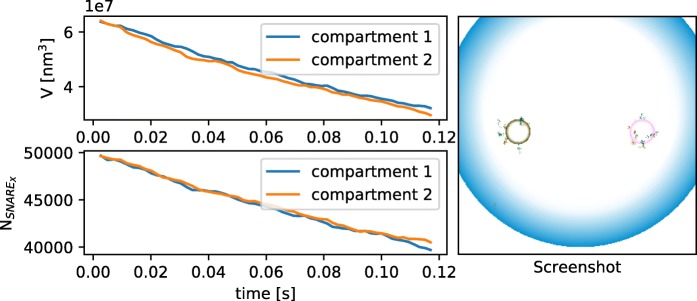
Volume and amount of SNARE _*X*_ over time and snapshot from the vesicle transport model. In the case of pure brownian motion the volume of the compartments decreases and no sorting of SNAREs takes place as shown on the left. The right side shows a screenshot from the simulation where you can see the random movement of the vesicles. The blue sphere is the cell with a radius of 2.5 *μm*

To show that the directed motion is essential for the sorting, as a control we run the simulation without the external force field and with compartments with equal initial size (*V*_1/2_≈ 0.065 *μm*^3^) and equally initially distributed SNAREs ($N_{1/2}^{X/Y}$ = 50000). In a purely Brownian model, i.e., a model where all particles perform Brownian motion, no sorting takes place and both compartments are just shrinking as shown in Fig. 5. It can also be seen that the vesicle’s slowly diffusion around the compartments and there behavior is independent of their coating. In contrast the vesicles in the model with a directed movement either re-fuse with the compartment of origin or move to the other compartment based on their coating. As a result vesicles in this model fuse with a specific compartment after a short time and by this they are sorting the SNAREs. This simplified model illustrates how external forces can simulate the directed motion in a biological system.

### The yeast model

A more complex model that showcases more of ML-Force’s abilities is based on an illustrative yeast model [30], which in turn uses the model of the cell cycle proposed by TYSON [49]. In the original model each yeast cell can grow and contains five different proteins whose amounts oscillate periodically and trigger the fission of a yeast cell. Each cell has a mating type (P or M) which can change during the cell fission. Depending on the type the yeast releases pheromones (M-factor and P-factor, respectively) to inhibit the cell cycle of cells with opposite mating type. In addition, the type M cells secrete a protease called Sxa2 which inactivates the P-factor pheromone that stems from the type P cells.

**Fig. 6:**
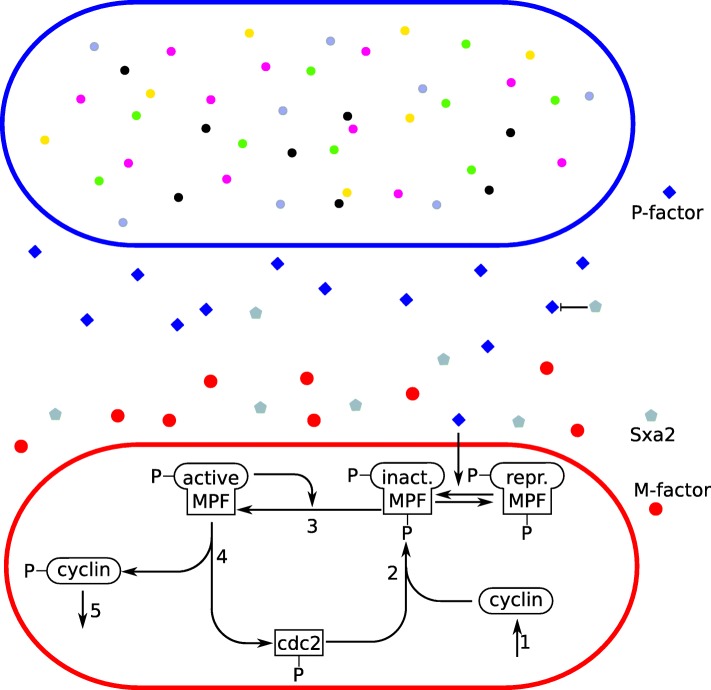
Sketch of the spatial/non-spatial cell cycle. Cell cycle of a yeast cell with five/six different proteins. The upper cell shows the cell cycle modeled with particles. As the proteins interaction within the cell occurs on a vastly different timescale than the behavior of the cell this is not a feasible approach. Instead the internal dynamics are modeled by a non-spatial approach as shown at the bottom

With the ML-Force model, we want to check whether cells of identical mating types accumulate in some areas and inhibit the cell cycle of cells with opposite mating type in this area. In ML-Force, each species of the system can be modeled as a particle, as shown in the upper part of Fig. 6. The problem of this approach are the different time scales of the dynamics. The cell cycle lasts about 120 min, while the diffusion of the proteins needs a time step in the sub nanosecond regime. In addition, for our question, a spatial simulation of the intra-cellular dynamics is not required. Therefore, we represent the pheromones and the cells as particles and the proteins involved in the cell cycle (as well as the cell cycle) as attributes of the cells, as shown in the lower part of Fig. 6. Whereas cells only move during the fission process in the *x*-*y* plane, pheromones and Sxa2 diffuse in the extracellular medium and disappear when they reach the borders of the test volume.

**Fig. 7:**
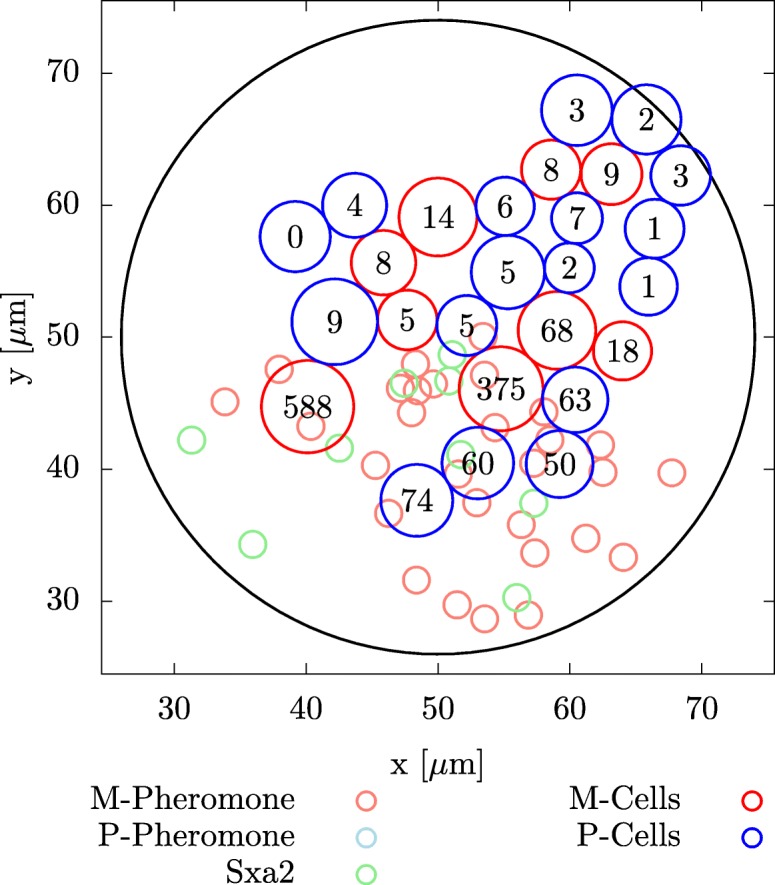
Spread of yeast cells. Distribution of yeast cells after 900 min. The numbers in the cells indicate how many pheromones are bound to them. For example the M-type cell on the left site has a highly inhibited cell cycle with 568 bounded pheromones. It is very likely that this cell will enter apoptosis, before it divides

As shown in Fig. 7 after 900 min there are twice as many P-type cells as M-type cells. Furthermore some of the M-type cells are old (large) and have a highly inhibited cell cycle, which makes it likely that these cell will enter apoptosis before they can divide.

Attributed particles and arbitrary functions allow to model the intra-cellular dynamics non-spatially as first order reaction. 
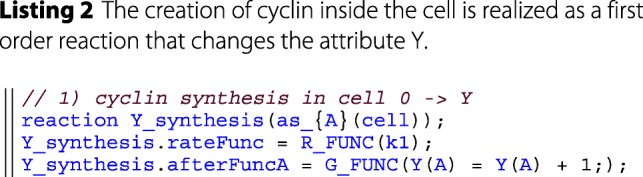


Nevertheless these non-spatial dynamic can influence the particle, here by triggering the division of a cell. 
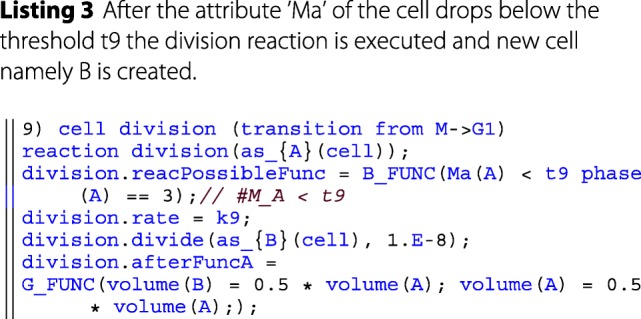


It is also possible for a particle to influence the attributes (non-spatial) of particle.


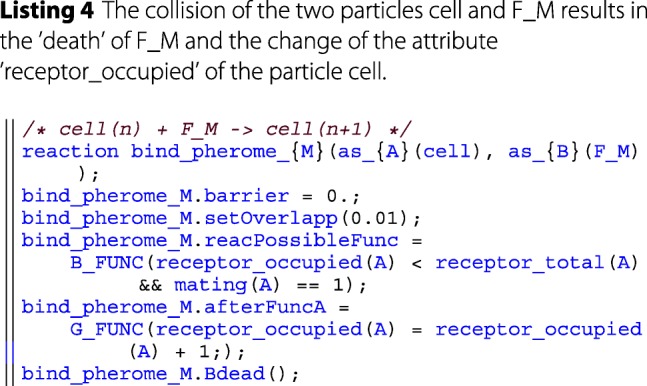


For example this is the case in the yeast model when a pheromone reacts with a cell (both particles) and inhibits the cell cycle of the cell. This combination of spatial and non-spatial behaviors enables the simulation of systems which include processes on different temporal scales.

### Lipid rafts model

The role of lipid rafts in inducing and promoting receptor accumulation within the cell membrane and the recruitment and binding of proteins from the cytosol has been subject to several computational studies [50, 51]. A small model of receptor lipid raft interaction which is inspired by a sub-model of Wnt signaling [52] shall illustrate the ability of ML-Force to support dynamic nesting. The model consists of two proteins (LRP 5/6 and CK1- *γ*) which diffuse in the membrane and may enter or leave lipid rafts. In the model, the mobility of proteins is reduced in lipid rafts by a 10 times larger viscosity in the rafts compared to the rest of the membrane. Both proteins have different affinities to enter the lipid raft which is modeled by different energy barriers in the nesting reactions. LRP 5/6 has to pass a barrier of 12·10^−21^ J which was estimated from the distribution of the kinetic energy for demonstration purposes. It shows how the energy barrier at a bimolecular reaction can be used to slow down the reaction. CK1- *γ* can enter the lipid raft freely (*E*_*barrier*_ = 0 J). By choosing a larger energy barrier for LRP 5/6 while they are entering the lipid raft we model their lower affinity to the lipid raft and can show how the energy barrier at a bimolecular reaction can be used to slow down the reaction. To show how these functions influence the accumulation of the proteins in the lipid raft we run a 2D simulation. In this simple experiment, our model contains a single lipid raft which covers 25% of the surface. The 10 times larger viscosity of the lipid raft causes a 10 times lower diffusion coefficient in the raft compared to the rest of the membrane. The simulation starts with 200 LRP 5/6 and 200 CK1- *γ* particles outside the lipid raft. To enter the lipid raft, the particles need to overcome the energy barriers described above. After a particle entered a lipid raft they can leave it again without any energy barrier.

**Fig. 8:**
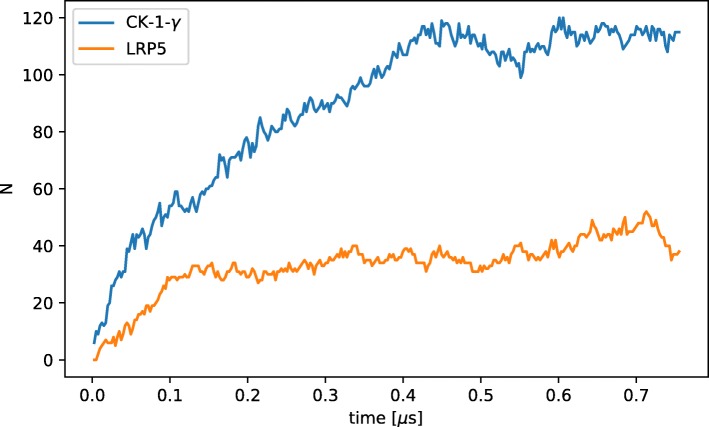
Accumulation of proteins in lipid rafts. Amount of LPR 5/6 and CK1- *γ* inside the lipid raft. Due to the lower affinity of LRP 5/6 to the lipid raft (realized by an energy barrier) their concentration is lower then the one of CK1- *γ*

As expected, Fig. 8 shows that due to the slower diffusion in the lipid raft CK1- *γ* accumulates in the lipid raft. The amount of LRP 5/6 which accumulates in the lipid raft is lower due to the energy barrier which hampers receptors to enter the lipid raft.

The model shows how the repulsive force during a bimolecular reaction can lower the reaction rate. Also the dynamic nesting of particles can change their behavior (here the diffusion of the nested particle) during simulation. It is also possible to restrict reactions to only occur if particles are nested inside a specific type of particle, such as the phosphorylation of LRP 5/6 which is constrained to lipid rafts.

## Discussion

The benchmark models which range from the multicellular yeast model to the subcellular lipid raft model have shown the usefulness of the realized features. ML-Force expands upon the state of the art. The combination of compartmental dynamics and particle simulation based on forces is a unique feature of ML-Force. This feature has been used in the lipid raft model (“Lipid rafts model” section). ML-Force provides a consistent force-based semantics for spatially resolved dynamic nesting with excluded volumes. Arbitrary functions and attributes have proven a necessity for a concise and computationally feasible realization of the yeast model in ML-Force (“The yeast model” section) where they are used for the non-spatial stochastic simulation of the cell cycle. In ML-Force, intra-particle dynamics can be simulated either spatially resolved by nested particles or in a non-spatial stochastic manner. Applying lower and higher spatial resolutions on demand facilitates modeling and simulating complex spatial models. The vesicle transport model (“A model of vesicular transport” section) relied on the possibility to let particles grow and to define global force functions that apply to all particles independently of other particles to simulate the directed movement of vesicles. The global force functions provide an additional means for the modeler for abstraction (in this case from the cytoskeleton structure on which the motor-proteins move). Along all other dynamics this is seamlessly integrated into the force-based semantics of ML-Force.

## Conclusions

The particle-based modeling and simulation approach ML-Force combines excluded volumes and forces with support for dynamic nesting (compartmental dynamics) and the ability of constraining cellular dynamics by arbitrary attributes and functions. Whereas other simulation approaches have treated compartmental dynamics, such as particles entering or leaving a compartment, or compartmental fission or fusion, discretely, in ML-Force those are simulated in a smooth, continuous manner. Thereby, structural dynamics are seamlessly integrated into the particle-based simulation governed by the LANGEVIN equation. However, this adds to the requirements the integrator has to face and consequently the induced calculation efforts.

ML-Force utilizes a rule-based modeling approach. In combination with attributed species of arbitrary types and arbitrary functions that work on particles, their attributes and content, this contributes to the expressiveness and flexibility of ML-Force. In particular, part of a spatial model can be executed in a non-spatial stochastic manner. Abstracting from spatial details on demand reduces the complexity of the model and the induced calculation effort and allows applying ML-Force to a wider range of cell biological systems. The possibility to define force functions that act on all particles independently of others provides another valuable means for abstraction in ML-Force.

Currently, ML-Force is being used in a combined in-vitro and in-silico study to analyze the impact of external electrical fields on cellular membranes. Future work will be aimed at enhancing the readability of ML-Force models by a more declarative expression of rules. In addition, user support for selecting time steps and suitable parameters for force calculation needs to be provided. Also advanced numerical integration schemes that are exploited by other particle-based simulators and their impact on compartmental dynamics shall be analyzed.

## Supplementary information

**Additional file 1** The additional file contains tests of of basic functions and the code of the models from the case study.

## Data Availability

The datasets used and/or analysed during the current study are available from the corresponding author on reasonable request.

## Abbreviations

CK1- *γ*: casein kinase 1- *γ*
DSL: domain specific language
LRP5/6: low-density lipoprotein receptor-related protein 5/6
ML: multi-level
RDME: reaction diffusion master equation
SNARE: soluble N-ethyl-maleimide–sensitive factor attachment protein receptors
