# Supplementary material for: Potential based, spatial simulation of dynamically nested particles
Source: BMC Bioinformatics. 2019 Nov 27;20:607. doi: 10.1186/s12859-019-3092-y (PMC6880518; doi:10.1186/s12859-019-3092-y)
Supplement: Supplementary file 1 — Additional file 1 The additional file contains tests of of basic functions and the code of the models from the case study. [file 12859_2019_3092_MOESM1_ESM.pdf]

1 Supplementary material - Additional tests

To test the accuracy of ML-Force simulations, we applied ML-Force to a small set of simple benchmarks.

1.1 Create reaction

The most simple and fundamental zero order reaction is the creation of a particle with a constant rate ($\emptyset \xrightarrow{\text{k}} A$). The reaction can be represented by the differential equation

$$\frac{dA}{dt} = k \tag{1}$$

which can easily be solved with the initial condition $A(t = 0) = 0$ and yields the solution $A(t) = kt$. To compare the results of ML-Force to this theoretical solution, we run 120 simulations with a creation rate of $k = 0.5 \frac{1}{\text{ns}}$ (see fig. 1). In each time step a exponential distributed random number is generated and compared with the rate times the time step. If the random number is larger or equal $k \cdot \Delta t$, the create reaction is executed and a new particle is created. We fitted the reaction rate to the simulation results. The fitted rate is 0.4975 $\frac{1}{\text{ns}}$ and therewith only differs by $\approx 0.5\%$ from the given rate, which is in good agreement with respect to the stochastic nature of the process.

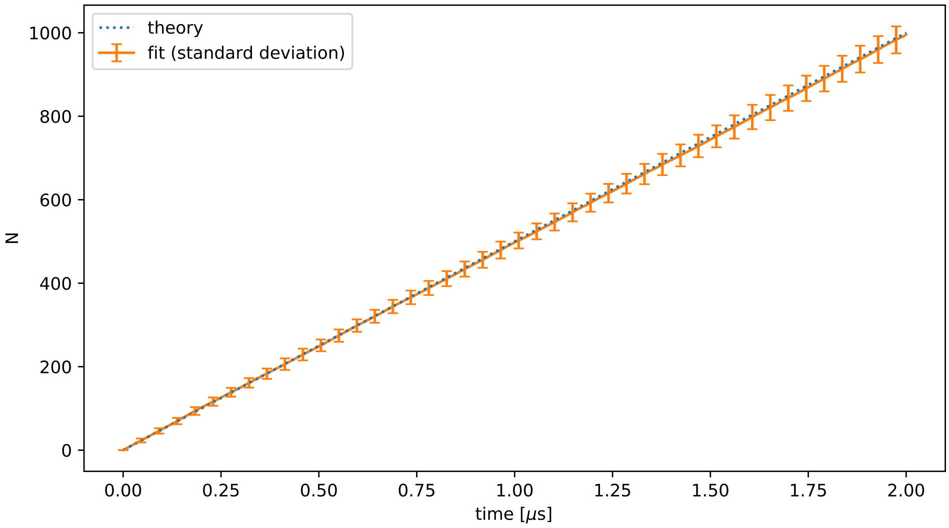

Figure 1: **Particle creation** Number of particle A over time for 120 runs with a creation rate of 0.5 $\frac{1}{\text{ns}}$. The lines show the linear fitted and the theoretically behavior. The vertical bars indicate the standard derivation of the simulation results.

Listing 1: Model for the test of the create function (create_test.cpp) that includes three species. *A* are the particles which are created. *Dummy* is a dummy for the calculation of the time step at the beginning of the simulation. The current prototypical implementation requires moving particles in the system to calculate the time step. *Box* is a large, fixed particles in the middle of the system, where the particles are created. One reaction (create) is defined in the system that simply adds a particle into *Box* with a given rate.

```
// 3D model
#include "simulator/dsl.h"
using namespace dsl;
namespace model {

void create_test() {
  double box_size = 1100 * units::nano_meter;
  set_temperature(300 * units::kelvin);
  set_viscocity(1E-10 * units::kilogram
          / units::nano_meter / units::second);
  setSize(box_size);

/* declaring species and properties */
  ADD_SPECIES(A);
  A.local_density = nature::protein_density;
  radius(A) = 5 * units::nano_meter;
  colorType(A) = 8;

  ADD_SPECIES(Dummy); // dummy partilce for the timestep
  Dummy.local_density = nature::protein_density;
  radius(Dummy) = 5 * units::nano_meter;
  colorType(Dummy) = 1;

  ADD_SPECIES(Box);
  Box.local_density = nature::protein_density;
  radius(Box) = 500 * units::nano_meter;
  colorType(Box) = 2;
  Box.move = false;

/* declaring Box[] -> Box[A] reaction */
  reaction create(as_A(Box));
  create.rate = 5.*1E8 / units::second;
  create.createIn_A(A);

/* inital state */
  putHere(Box, box_size / 2, box_size / 2, box_size / 2);
  putSomewhere(Dummy, 2);

/* defines output */
  observe::nr_of(A);

  visualize(25);
  }
}
```

1.2 Decay reaction

As a second test we simulate the decay of particles in ML-Force. The decay of particles is described by the first order reaction $A \xrightarrow{\text{k}} \emptyset$

$$\frac{dA}{dt} = -k \cdot A \tag{2}$$

With the initial condition $A(t = 0) = A_0$ we get the theoretical solution $A(t) = A_0 e^{-kt}$. In ML-Force 100 simulation runs were executed with a decay rate constant of $k = 0.5 \frac{1}{\text{s}}$ and starting with 1000 particles. The fitted decay rate over 100 runs is 0.499973 $\frac{1}{\text{s}}$ and therewith only differs by $\approx 0.0054\%$ from the given rate, as shown in fig. 2. Again the ML-Force simulator samples from an exponential distribution with $\lambda = 1$ and compares it with $k \cdot \Delta t$ to determine if the particle decays. Since this is a first order reaction the sampling is executed for each particle individually. Therefore, the good agreements of the two first tests to theoretical results are not surprising.

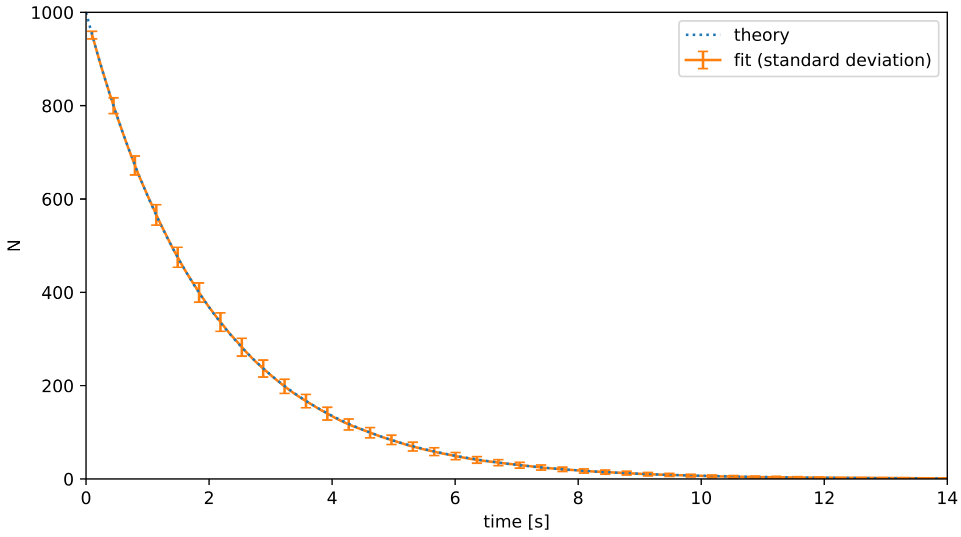

Figure 2: **Particle decay** Number of particle A over time for 100 runs with a decay rate of 0.5 $\frac{1}{\text{s}}$ and $A_0 = 1000$ particles. The lines show the fitted and the theoretically behavior and the vertical bars indicate the standard derivation of the simulation results.

Listing 2: Model for the test of the decay function (decay_test.cpp). There is only one species in it, the particle A. This species can undergo the reaction decay, that removes A from the system with a defined rate.

```
// 3D model
```

```
#include "simulator/dsl.h"
using namespace dsl;
namespace model {

void decay_test() {
  double box_size = 1100 * units::nano_meter;
  set_temperature(300 * units::kelvin);
  set_viscocity(1E-10 * units::kilogram
    / units::nano_meter / units::second);
  setSize(box_size);

/* declaring species and properties */
  ADD_SPECIES(A);
  A.local_density = nature::protein_density;
  radius(A) = 5 * units::nano_meter;
  colorType(A) = 8;

/* declaring A -> 0 reaction */
  reaction decay(as_A(A));
  decay.rate = 0.5 / units::second;
  decay.Adead();

/* inital state */
  putSomewhere(A, 1000);

/* defines output */
  observe::nr_of(A);

  visualize(25);
  }
}
```

1.3 Second order reactions

As mentioned in sec. 2.2.3 of the main text the macroscopic reaction rate distinguish two processes

1. diffusion limit: Every collision of two reactants yields to a reaction. In this case the 3D rate is described by the SMOLUCHOWSKI rate [3]

$$k(t) = 4\pi\hat{D}\sigma\left[1 + \frac{\sigma}{\sqrt{\pi\hat{D}t}}\right] \quad , \tag{3}$$

where $\hat{D}$ is the sum of the diffusion coefficients ($\hat{D} = D_A + D_B$), that are calculated from the STOKES-EINSTEIN equation (see sec. 1.4) and σ is the sum of the radii ($\sigma = r_A + r_B$) of the particles. In the steady state ($t \to \infty$) the rate becomes

$$k = 4\pi\hat{D}\sigma. \tag{4}$$

2. activation limit: In this case not every collision of two reactants results in a reaction. The steady state rate in 3D is given by the COLLINS and KIMBALL model [1]

$$k = \left(\frac{1}{4\pi \hat{D} \sigma} + \frac{1}{k_{int}} \right)^{-1} \quad , \tag{5}$$

where k_{int} is the intrinsic rate. For an infinite intrinsic rate the COLLINS and KIMBALL rate transfers to the steady state SMOLUCHOWSKI rate.

In ML-Force we tested for the diffusion limited cases of an irreversible reaction ($A + A \to \emptyset$) in 3D and 2D and the reversible reaction ($A + B \leftrightarrow C$) in 3D.

1.3.1 Irreversible reaction in 3D

First we test the irreversible reactions in the diffusion-limited regime in 3D in ML-Force. We initialize a system with 2000 randomly distributed particles of type A which are placed in a box with a volume of ≈ 200000 nm^3. These particles undergo an annihilation reaction ($A + A \to \emptyset$) with the SMOLUCHOWSKI rate (eq. 3). We receive the differential equation of the density

$$\frac{d\rho_A}{dt} = -k(t) \cdot \rho_A^2. \tag{6}$$

With the initial condition $\rho_A(t = 0) = \rho_{A0}$, we get the solution

$$\rho_A(t) = \left(\frac{1}{\rho_{A0}} + 4\pi \hat{D} \sigma t \right)^{-1} \tag{7}$$

for the steady state rate and

$$\rho_A(t) = \left(\frac{1}{\rho_{A0}} + 4\pi \hat{D} \sigma t + 2\sigma^2 \sqrt{\pi \hat{D} t} \right)^{-1} \tag{8}$$

for the time dependent rate.

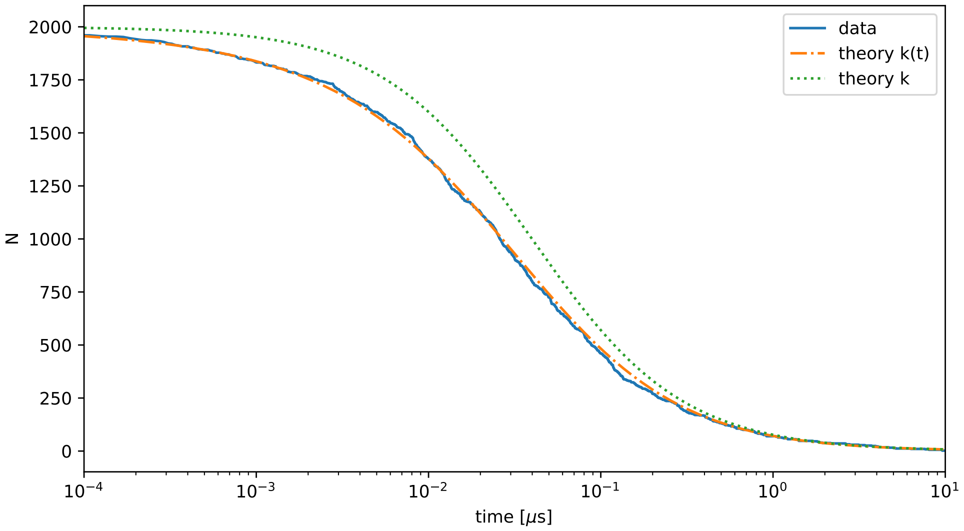

Figure 3: **Irreversible particle reaction in 3D** The blue line shows the simulation results and the orange and green line are the theoretical results for the time dependent and steady-state rate respectively.

The results (fig. 3) show that the reaction rate is time dependent and in good agreement with the theory.

Listing 3: Model for the test of the irreversible reaction in 3D (irrev_react.cpp). There is only one species in the model, the particle part_A. This species can undergo the reaction irrev, that removes two part_A from the system if they collide. The energy barrier between both reactants is set to zero, to simulate a diffusion limited reaction. The function setOverlapp(d) ($d \in [0; 1]$) defines which ration of the diameter of the smaller particle have to be inside the larger one in order to execute the reaction rule.

```
// 3D model
#include "simulator/dsl.h"
using namespace dsl;
namespace model {

void irrev_react() {
  const double box_size = 58.5 * units::nano_meter;

  set_temperature(300 * units::kelvin);
  set_viscocity(4.392E-12 * units::kilogram
    / units::nano_meter / units::second);
  setSize(box_size);
```

```
    /* declaring species and properties */
    ADD_SPECIES(part_A);
    part_A.local_density = nature::protein_density;
    radius(part_A) = 0.5 * units::nano_meter;
    colorType(part_A) = 1;

    /* declaring A + A -> 0 reaction */
    reaction irrev(as_A(part_A), as_B(part_A));
    irrev.barrier = 0.;
    irrev.Adead();
    irrev.Bdead();
    irrev.setOverlapp(0.01);

    /* inital state */
    putSomewhere(part_A, 2000);

    /* defines output */
    observe::nr_of(part_A);

    visualize(25);
    }
}
```

1.3.2 Irreversible reaction in 2D

As a second test we use the same annihilation reaction in 2D. For the diffusion limited case the reaction rate [2, 4] is given by

$$k(t) = \frac{8D}{\pi} \int_0^\infty \frac{e^{-Du^2 t}}{u[Y_0^2(\sigma u) + J_0^2(\sigma u)]} du \tag{9}$$

where Y_0 and J_0 are the Bessel function of first and second kind. To test the 2D annihilation reaction we initialize a system with 200 randomly distributed particles of type A in a square with a surface of 10000 nm^2 and run 10 simulations. The results are shown in fig. 4.

The simulation is in acceptable agreement to the theory with respect to the stochastic nature of the particle-based approach.

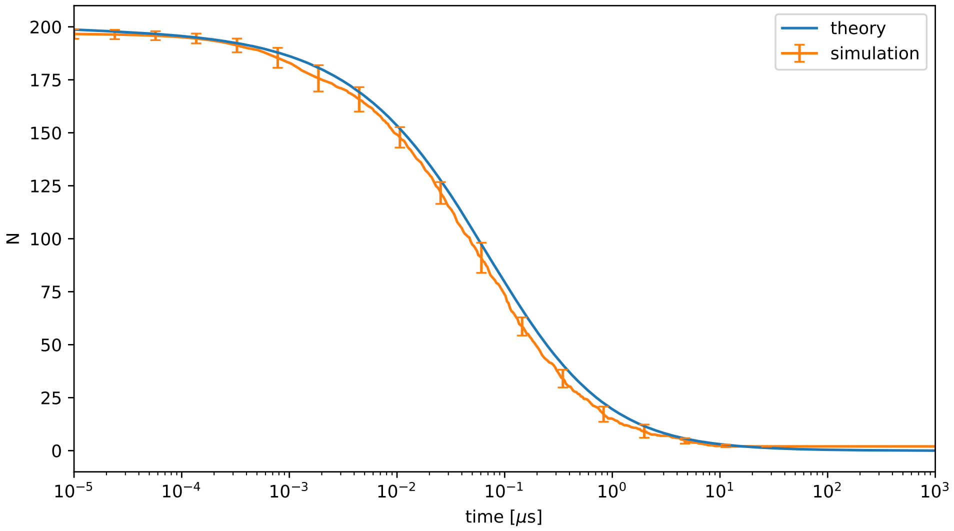

Figure 4: **Irreversible particle reaction in 2D** The blue line shows the theoretical number of particles for the 2D annihilation reaction. The orange line shows the mean number of particles from the 10 simulations. The vertical bars indicate the standard derivation of the simulation results.

Listing 4: Model for the test of the irreversible reaction in 2D (irrev_react_2D.cpp). As in the 3D version, the only particle part_A can undergo only the diffusion limited reaction irrev, that removes two part_A from the system if they collide. The function setOverlapp(d) (d $\in$ [0; 1]) defines which ration of the diameter of the smaller particle have to be inside the larger one in order to execute the reaction rule.

```
// 2D model
#include "simulator/dsl.h"
using namespace dsl;
namespace model {

void irrev_react_2D() {
  const double box_size = 1000 * units::nano_meter;

  set_temperature(300 * units::kelvin);
  set_viscocity(50 * 4.392E-12 * units::kilogram / units::nano_meter /
                units::second);
  setSize(box_size);

  /* declaring species and properties */
  ADD_SPECIES(part_A);
```

```
    part_A.local_density = nature::protein_density;
    radius(part_A) = 0.5 * units::nano_meter;
    colorType(part_A) = 1;

    /* declaring A + A -> 0 reaction */
    reaction irrev(as_A(part_A), as_B(part_A));
    irrev.barrier = 0.;
    irrev.Adead();
    irrev.Bdead();
    irrev.setOverlapp(0.01);

    /* inital state */
    putSomewhere(part_A, 200);

    /* defines output */
    observe::nr_of(part_A);

    visualize(25);
  }
}
```

1.3.3 Reversible reaction

Reversible reactions (like $A + B \leftrightarrow C$) are much harder to handle in a particle-based approach than irreversible reaction. The reason is that after the dissociation of particle C ($C \to A + B$) the resulting particles A and B are located nearby and hence it is very likely that they react back to C, which results in a wrong equilibrium (see figure 5). In ML-Force, the particles A and B are placed at the position of the disassociated particle C and separated by a force which moves both apart from each other until they do no longer overlap. This force is inspired by the potential energy surface of a chemical reaction. During the reaction the reactants form an activated complex with a potential energy equal to the activation energy. From this state the system changes to an energetically lower state and the potential energy dissipates into the system as heat. When a particles splits into two, the change into a energetically lower state also changes the distance between the particles. Since this process serves merely as an inspiration for the separation force, the force itself cannot be derived from the system but is found in a trial and error fashion. The separation force is calculated from the mass of the particles and a predefined acceleration. By a proper choice of the acceleration, the right equilibrium can be reached. If the force is too small, the system will end in a wrong equilibrium. Is the force too large, the particles will be accelerated too much and reach a unrealistic high velocity which can be seen by comparing the simulated velocity of the particles to the theoretical one from the Maxwell-Boltzmann distribution.

To demonstrate the use of the separation force, we use a system with 1000 particles of type A and B. The rate of reaction $A + B \to C$ follows the SMOLUCHOWSKI rate whereas the reaction $C \to A + B$ occurs with a rate of $k = 4390352\ \mathrm{s}^{-1}$ which should lead to an equilibrium with A=B=C=500.

As shown in fig. 5 for a sufficient strong separation force the equilibrium differs just

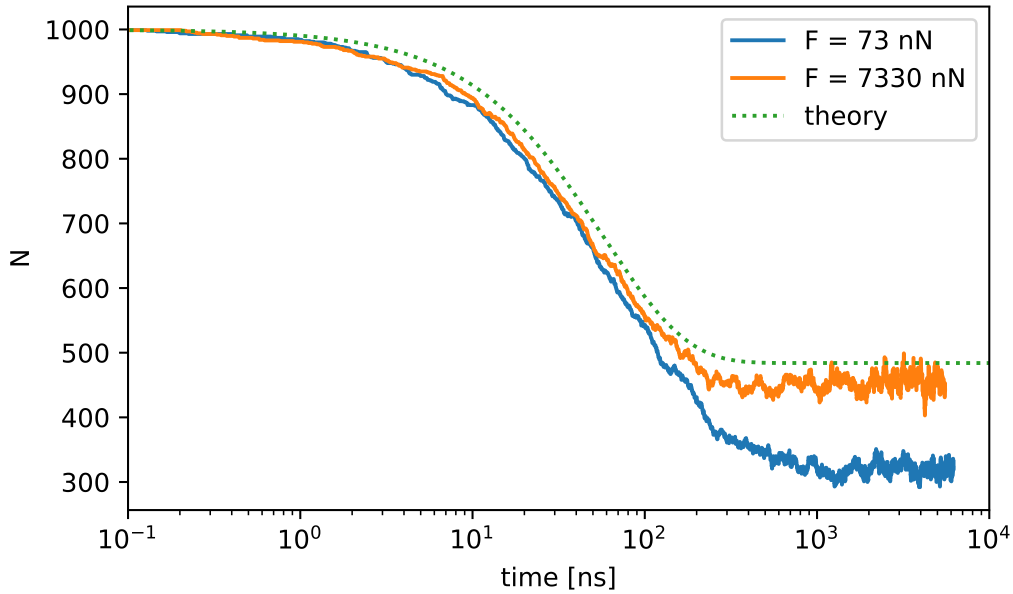

Figure 5: **Reversible particle reaction** Number of particle A for the reversible reaction $A + B \leftrightarrow C$ for two different separation forces.

slightly from the theoretical results.

Listing 5: Model for the test of the reversible reaction (rev_react.cpp). There are three species in the system: part_A, part_B and part_C. The species part_A and part_B can undergo the reaction dimer, where they form the particle part_C if they collide. The energy barrier between both particle is set to zero to simulate a diffusion limited reaction. The function setOverlapp(d) defines how much the particles have to overlap for the reaction to start. The species part_C can undergo the reaction decay_mol, where part_C is divide into part_C and part_B (divide) and then part_C is changed to part_A (afterFuncA). The acceleration that is given in the divide function is used to calculate the force that separates the both particles that are created during the division.

```
// 3D model
#include "simulator/dsl.h"
using namespace dsl;
namespace model {

void rev_react() {
  set_temperature(300 * units::kelvin);
  set_viscocity(4.392E-12 * units::kilogram
    / units::nano_meter / units::second);
```

```
  const double box_size = 58.5 * units::nano_meter;

/* declaring species and properties */
  ADD_SPECIES(part_A);
  radius(part_A) = 0.5 * units::nano_meter;
  colorType(part_A) = 1;
  part_A.local_density = nature::protein_density;

  ADD_SPECIES(part_B);
  radius(part_B) = 0.5 * units::nano_meter;
  colorType(part_B) = 3;
  part_B.local_density = nature::protein_density;

  ADD_SPECIES(part_C);
  radius(part_C) = 0.5 * units::nano_meter;
  colorType(part_C) = 6;
  part_C.local_density = nature::protein_density;

  setSize(box_size);

/* declaring A + B -> C reaction */
  reaction dimer(as_A(part_A), as_B(part_B));
  dimer.barrier = 0.;
  dimer.afterFuncA =
      G_FUNC(changeSpecies(A, part_C); radius(A) = 0.5 * units::nano_meter;);
  dimer.Bdead();
  dimer.setOverlapp(0.3);

/* declaring C -> A + B reaction */
  reaction decay_mol(as_A(part_C));
  decay_mol.rate = 4390352 / units::second;
  decay_mol.divide(as_B(part_B),
    5.E25 * units::nano_meter / units::second / units::second);
  decay_mol.afterFuncA =
    G_FUNC(changeSpecies(A, part_A);
    radius(A) = 0.5 * units::nano_meter;);
  decay_mol.afterFuncB = G_FUNC(radius(B) = 0.5 * units::nano_meter;);

/* inital state */
  putSomewhere(part_A, 1000);
  putSomewhere(part_B, 1000);

/* defines output */
  observe::nr_of(part_A);
  observe::nr_of(part_B);
  observe::nr_of(part_C);

  visualize(25);
  }
}
```

1.4 Diffusion

In ML-Force models, the diffusion coefficient is not explicitly specified. Instead it is calculated from the STOKES-EINSTEIN equation

$$D = \frac{k_B T}{6\pi\eta r} = \frac{k_B T}{\gamma} \tag{10}$$

for each particle where γ is the friction coefficient, which results from the viscosity η and the radius r of the particle. In theory diffusion is described by Fick's second law

$$\frac{\partial\rho}{\partial t} = D\frac{\partial^2\rho}{\partial x^2} \tag{11}$$

where ρ is the concentration of particles. The solution of the equation is given by

$$\rho(x,t) = \frac{N}{\sqrt{4\pi Dt}} \exp\left(-\frac{(x-x_0)^2}{4Dt}\right) \tag{12}$$

for the case of an infinite large space and a point source at x_0. These initial condition are described by $\rho(x, t = 0) = N \cdot \delta(x - x_0)$ where N is the number of particles and $\delta(x)$ is the delta distribution. This initialization means, that N particles without a volume (point particles) are located at the position x_0 which is a common initial condition for PDEs.

To test the diffusion in ML-Force we initialize particles with the distribution $\rho(x_0, t = 1\mu s)$ and let them diffuse for 15 μs. We choose the time t=1 μs instead of t = 0 μs since it is not possible to initialize a point source in a particle based simulator with excluded volumes. To avoid the effects of a crowded environment we only put 20 particles in the system and simulate it 50 times. Furthermore the diffusion coefficient is isotrop (independent of the direction) and we can simulate the system in 3D and compare the resulting position of the particles to the theoretical 1D distribution. This gives as $20 \cdot 50 \cdot 3 = 3000$ positions for $t_0 = 1$ μs and $t_{end} = 16$ μs. These values where plotted in a histogram and a normal distribution fitted to them. From the mean and standard derivation of $x_{0,fit} = \mu_0$ and $D_{fit} = \sigma^2/2t$. We have simulated the diffusion for two different time steps (granularity g = 150 and g = 20, see Sec. integrator of the main text) and the results are shown in fig. 6.

For the smaller time step (g = 150) the means of the distributions are 499.9 nm and 499.3 nm for t = 1 μs and t = 16 μs, respectively ($x_{0,theo}$ = 500 nm). Since the mean of the distribution only shifts slightly there is no directed movement in addition to the diffusion. For the larger time step (g = 20) the means are 499.7 nm and 496.6 nm for t = 1 μs and t = 16μs, respectively. So also for the larger time step the particles diffuse without a drift since the shift of 3.1 nm is small compared to the broadening of the distribution and the noise. Unlike the lack of a drift to the diffusion, the diffusion coefficient is sensitive to the time step. The theoretical diffusion coefficient is 100.1 $\frac{nm^2}{\mu s}$. For the smaller time step (g = 150) the D_{fit} is 102.4 $\frac{nm^2}{\mu s}$ at t = 1 μs and 106.3 $\frac{nm^2}{\mu s}$ at t = 16 μs. In comparison the D_{fit} is 98.6 $\frac{nm^2}{\mu s}$ at t = 1 μs and 321.4 $\frac{nm^2}{\mu s}$ at t = 16 μs for

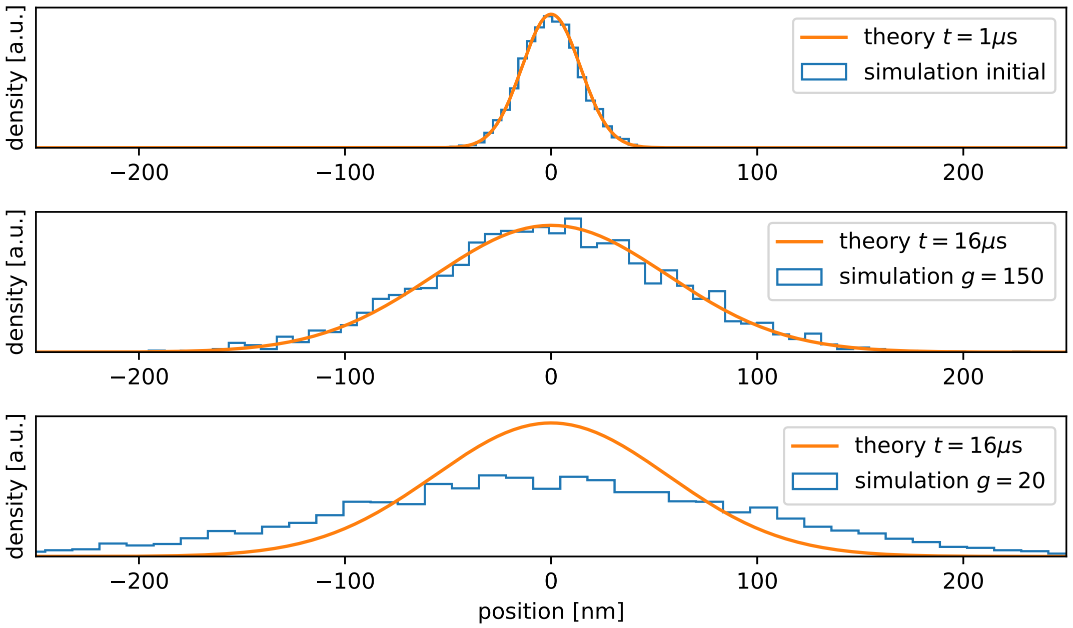

Figure 6: **Particle diffusion** Distribution of particles from the simulation (blue bins) and the theory (orange line). The plot at the top show the initial distribution $\rho(x_0, t = 1\mu s)$. The plots at the middle and bottom show the distribution after the particles propagated for 15 μs for a granularity of g = 150 and g = 20, respectively.

the larger time step (g = 20). The reason for the error in sampling the diffusion is due to the finite friction in the system. During the propagation of a particle it constantly looses energy by friction and gets new energy by collisions with the solvent molecules. By using a large time step the integration error of the friction produces a smaller loss of energy by friction and results in a higher velocity of the particles. This shows the importance of using a sufficient small time step in ML-Force.
Note: All the calculation were also done for the x, y and z coordinates separately to ensure that there are no contrary shifts that cancel out in the sum of the data. This was not the case. Since the diffusion coefficient is the same in each direction the diffusion is indeed isotrop as assumed at the beginning.

Listing 6: Model for testing the diffusion (diff_test.cpp). Only one species (A) is contained in the system. 20 particles are placed in the middle of the simulation volume with the initial concentration $\rho(x = x_0, t = 1\mu s)$ and diffuse without any reactions.

```
// 3D model
#include "simulator/dsl.h"
```

```
#include <random>
#include <cmath>
#include <ctime>

using namespace dsl;

namespace model {

void diff_test() {

  double box_size = 1100 * units::nano_meter;
  set_temperature(300 * units::kelvin);
  set_viscocity(4.392E-12 * units::kilogram / units::nano_meter /
                units::second);
  setSize(box_size);

  /* declaring species and properties */
  ADD_SPECIES(A);
  A.local_density = nature::protein_density;
  radius(A) = 0.5 * units::nano_meter;
  colorType(A) = 8;

  double D;
  D = nature::boltzmann * 300 /
      (6 * mPI * 4.392E-12 * units::kilogram / units::nano_meter /
       units::second * 0.5 * units::nano_meter);
  double x0 = 500;
  const unsigned int seed = time(0);

  std::mt19937 gen{ seed };
  std::normal_distribution<> d{ 0, 1 };

  for (int i = 1; i <= 20; i++) {
    putHere(A, sqrt(2. * D * 1 * units::micro * units::second) * d(gen) + x0,
            sqrt(2. * D * 1 * units::micro * units::second) * d(gen) + x0,
            sqrt(2. * D * 1 * units::micro * units::second) * d(gen) + x0);
  };

  visualize(25);
}
}
```

The above tests also emphasize ones more that the correct use of particle based simulators strongly depends on simulation parameters such as time steps but also on model parameters such as separating acceleration. As these parameters need to be determined for each concrete model new, computational support which exploits tests such as the ones above to automatically determine suitable simulation and model parameters appears therefore a prerequisite for a wider adaptation of particle-based modeling and simulation approaches in cell biology.

2 Main models

In this section the models from the case study of the main text are shown.

2.1 A model of vesicular transport

Listing 7: Model to simulate the vesicle transport from SNAREs between two compartments with a directed movement (vesicular.cpp). For the case of a pure Brownian motion the functions add_force have to be deleted.

```
// 3D model
#include "simulator/dsl.h"
using namespace dsl;
namespace model {

void vesicular() {
  set_temperature(300 * units::kelvin);
  set_viscocity(4.392E-12 * units::kilogram
    / units::nano_meter / units::second);

  const int amount_X = 2 * 50000;
  const int amount_Y = 2 * 50000;
  const int box_size = 5200 * units::nano_meter;

  ADD_SPECIES(cell);
  radius(cell) = 2500 * units::nano_meter;
  colorType(cell) = 55;
  cell.move = false;
  cell.local_density = nature::protein_density;

  const double r_comp = 250 * units::nano_meter;
  const double v_comp = 3.14 * 4 / 3 * pow(r_comp, 3);

  ADD_SPECIES(comp_1);
  volume(comp_1) = 2 * v_comp * 0.9;
  colorType(comp_1) = 3;
  comp_1.move = false;
  auto ADD_PROPERTY(snare_X, comp_1, int, amount_X * 0.9);
  auto ADD_PROPERTY(snare_Y, comp_1, int, amount_Y * 0.9);
  comp_1.local_density = nature::protein_density;

  ADD_SPECIES(comp_2);
  volume(comp_2) = 2 * v_comp * 0.1;
  colorType(comp_2) = 4;
  comp_2.move = false;
  ADD_PROPERTY(snare_X, comp_2, int, amount_X * 0.1);
  ADD_PROPERTY(snare_Y, comp_2, int, amount_Y * 0.1);
  comp_2.local_density = nature::protein_density;

  const double r_ves = 25 * units::nano_meter;

  ADD_SPECIES(ves_A);
```

```
  radius(ves_A) = r_ves;
  colorType(ves_A) = 1;
  ADD_PROPERTY(snare_X, ves_A, int, 0);
  ADD_PROPERTY(snare_Y, ves_A, int, 0);
  ves_A.local_density = nature::protein_density;
  ves_A.diffuse = true;

  ADD_SPECIES(ves_B);
  radius(ves_B) = r_ves;
  colorType(ves_B) = 2;
  ADD_PROPERTY(snare_X, ves_B, int, 0);
  ADD_PROPERTY(snare_Y, ves_B, int, 0);
  ves_B.local_density = nature::protein_density;
  ves_B.diffuse = true;

  ADD_SPECIES(dummy);
  radius(dummy) = r_ves;
  colorType(dummy) = 13;
  dummy.local_density = nature::protein_density;

  Vec pos_1;
  pos_1.at(0) = 0.5 * box_size + 0.25 * 2 * radius(cell);
  pos_1.at(1) = 0.5 * box_size - 0.07 * 2 * radius(cell);
  pos_1.at(2) = 0.5 * box_size;
  Vec pos_2;
  pos_2.at(0) = 0.5 * box_size - 0.25 * 2 * radius(cell);
  pos_2.at(1) = 0.5 * box_size - 0.07 * 2 * radius(cell);
  pos_2.at(2) = 0.5 * box_size;

 /* "dipole" like force to move the vesicle */

  add_force(
    F_FUNC(1E-21 * ((A.position - pos_1) / (pow(abs(A.position - pos_1), 3)) -
    (A.position - pos_2) / (pow(abs(A.position - pos_2), 3)))
    .normalize()),
    B_FUNC(A.type == ves_A.type));
  add_force(F_FUNC(-1E-21 *
    ((A.position - pos_1) / (pow(abs(A.position - pos_1), 3)) -
    (A.position - pos_2) / (pow(abs(A.position - pos_2), 3)))
    .normalize()),
    B_FUNC(A.type == ves_B.type));



/* budding of new vesicles from compartment 1 or 2 with coat A or B */
/* comp_1 -> comp_1 + ves_A */
  reaction budding_1_A(as_A(comp_1));
  budding_1_A.reacPossibleFunc =
    B_FUNC(volume(A) > 330000 * pow(units::nano_meter, 3));
  budding_1_A.rateFunc =
    R_FUNC(0.0001 * volume(A) / pow(units::nano_meter, 3) / units::second);
  budding_1_A.createAt_A(ves_A);
  budding_1_A.create_atA_func = C_FUNC(
    snare_X(created) = 500 * snare_X(A) / surface(A);
```

```
    snare_X(A) -= snare_X(created);
    snare_Y(created) = 500 * snare_Y(A) / surface(A);
    snare_Y(A) -= snare_Y(created);
    volume(A) = volume(A) - volume(created););

/* comp_1 -> comp_1 + ves_B */
  reaction budding_1_B(as_A(comp_1));
  budding_1_B.reacPossibleFunc =
    B_FUNC(volume(A) > 330000 * pow(units::nano_meter, 3));
  budding_1_B.rateFunc =
    R_FUNC(0.0001 * volume(A) / pow(units::nano_meter, 3) / units::second);
  budding_1_B.createAt_A(ves_B);
  budding_1_B.create_atA_func = C_FUNC(
    snare_X(created) = 50 * snare_X(A) / surface(A);
    snare_X(A) -= snare_X(created);
    snare_Y(created) = 50 * snare_Y(A) / surface(A);
    snare_Y(A) -= snare_Y(created);
    volume(A) = volume(A) - volume(created););

/* comp_2 -> comp_2 + ves_A */
  reaction budding_2_A(as_A(comp_2));
  budding_2_A.reacPossibleFunc =
    B_FUNC(volume(A) > 330000 * pow(units::nano_meter, 3));
  budding_2_A.rateFunc =
    R_FUNC(0.0001 * volume(A) / pow(units::nano_meter, 3) / units::second);
  budding_2_A.createAt_A(ves_A);
  budding_2_A.create_atA_func = C_FUNC(
    snare_X(created) = 500 * snare_X(A) / surface(A);
    snare_X(A) -= snare_X(created);
    snare_Y(created) = 500 * snare_Y(A) / surface(A);
    snare_Y(A) -= snare_Y(created);
    volume(A) = volume(A) - volume(created););

/* comp_2 -> comp_2 + ves_B */
  reaction budding_2_B(as_A(comp_2));
  budding_2_B.reacPossibleFunc =
    B_FUNC(volume(A) > 330000 * pow(units::nano_meter, 3));
  budding_2_B.rateFunc =
    R_FUNC(0.0001 * volume(A) / pow(units::nano_meter, 3) / units::second);
  budding_2_B.createAt_A(ves_B);
  budding_2_B.create_atA_func = C_FUNC(
    snare_X(created) = 50 * snare_X(A) / surface(A);
    snare_X(A) -= snare_X(created);
    snare_Y(created) = 50 * snare_Y(A) / surface(A);
    snare_Y(A) -= snare_Y(created);
    volume(A) = volume(A) - volume(created););

/* fusion of vesicles with compartment 1 or 2 */
/* comp_1 + ves_A -> comp_1 */
  reaction fusion_1_A(as_A(comp_1), as_B(ves_A));
  fusion_1_A.barrier = 0.;
  fusion_1_A.afterFuncA =
    G_FUNC(volume(A) = volume(A) + volume(B);
    snare_X(A) += snare_X(B);
```

```
    snare_Y(A) += snare_Y(B););
  fusion_1_A.Bdead();

/* comp_2 + ves_A -> comp_2 */
  reaction fusion_2_A(as_A(comp_2), as_B(ves_A));
  fusion_2_A.barrier = 0.;
  fusion_2_A.afterFuncA =
    G_FUNC(volume(A) = volume(A) + volume(B);
    snare_X(A) += snare_X(B);
    snare_Y(A) += snare_Y(B););
  fusion_2_A.Bdead();

/* comp_1 + ves_B -> comp_1 */
  reaction fusion_1_B(as_A(comp_1), as_B(ves_B));
  fusion_1_B.barrier = 0.;
  fusion_1_B.afterFuncA =
    G_FUNC(volume(A) = volume(A) + volume(B);
    snare_X(A) += snare_X(B);
    snare_Y(A) += snare_Y(B););
  fusion_1_B.Bdead();

/* comp_2 + ves_B -> comp_2 */
  reaction fusion_2_B(as_A(comp_2), as_B(ves_B));
  fusion_2_B.barrier = 0.;
  fusion_2_B.afterFuncA =
    G_FUNC(volume(A) = volume(A) + volume(B);
    snare_X(A) += snare_X(B);
    snare_Y(A) += snare_Y(B););
  fusion_2_B.Bdead();

/* Adding observations */
// time // 1
  observe::nr_of(ves_A);                          // 2
  observe::nr_of(ves_B);                          // 3
  observe::average_prop(ves_A, snare_X);   // 4
  observe::average_prop(ves_B, snare_X);   // 5
  observe::average_prop(comp_1, snare_X); // 6
  observe::average_prop(comp_2, snare_X); // 7
  observe::average_prop(ves_A, snare_Y);   // 8
  observe::average_prop(ves_B, snare_Y);   // 9
  observe::average_prop(comp_1, snare_Y); // 10
  observe::average_prop(comp_2, snare_Y); // 11
  observe::average_prop(comp_1, volume);  // 12
  observe::average_prop(comp_2, volume);  // 13

/* setting size of the system */
  setSize(box_size);

/* Adding objects to system */
  auto cell_1 = putHere(cell, 0.5 * box_size, 0.5 * box_size, 0.5 * box_size);
  putInsideAt(cell_1, comp_1, pos_1.at(0), pos_1.at(1), 0.5 * box_size);
  putInsideAt(cell_1, comp_2, pos_2.at(0), pos_2.at(1), 0.5 * box_size);
  putHere(dummy, 0, 0, 0);
  putHere(dummy, 0, 0, 0);
```

```
/* run the visualization */
  visualize(30);
  }
}
```

2.2 The yeast model

Listing 8: Model to simulate growth of yeast cells and inhibition of the cell cycle by pheromones (yeast_model.cpp).

```
// 3D model
#include "simulator/dsl.h"
using namespace dsl;
namespace model {
void yeast() {
  set_temperature(300 * units::kelvin);
  set_viscocity(3.5E-11 * units::kilogram / units::nano_meter / units::second);

  const double V_0 = 50 * 1E9 * pow(units::nano_meter, 3);
  const int Dtot_int = 1000;
  const double k1 = 0.015 / units::minute * 1000;
  const double k2 = 200 / units::minute;
  const double k3prime = 0.018 / units::minute;
  const double k3 = 680 / units::minute;
  const double k4 = 4.5 / units::minute;
  const double k5 = 0.6 / units::minute;
  const double k6 = 1 / units::minute;
  const double k7 = 1.E4 / units::minute;
  const double k8 = 1.E4 / units::minute;
  const double k9 = 1.E4 / units::minute;
  const double k10 = 1 / units::minute;
  const double k13 = 0.005 / units::minute;

  const double Td = 116;
  const int t7 = 250;
  const int t8 = 70;
  const int t9 = 20;
  const double box_size = (50000 + 50000) * units::nano_meter;

  ADD_SPECIES(Box);
  radius(Box) = 24000 * units::nano_meter;
  Box.move = false;
  Box.local_density = nature::water_density;
  Box.colortype = 5;

  ADD_SPECIES(F_M); // Pheromone M
  radius(F_M) = 1. * 1e-6 * units::meter;
  F_M.local_density = nature::protein_density;
  F_M.colortype = 8;

  ADD_SPECIES(F_P); // Pheromone P
```

```
  radius(F_P) = 1. * 1e-6 * units::meter;
  F_P.local_density = nature::protein_density;
  F_P.colortype = 12;

  ADD_SPECIES(X); // Sxa2
  radius(X) = 1. * 1e-6 * units::meter;
  X.local_density = nature::protein_density;
  X.colortype = 15;

  ADD_SPECIES(dummy);
  radius(dummy) = 1. * 1e-6 * units::meter;
  dummy.local_density = nature::protein_density;
  dummy.colortype = 45;

  ADD_SPECIES(cell); // yeast cell
  volume(cell) = V_0;
  cell.local_density = 1000 * nature::protein_density;
  colorType(cell) = 2;
  cell.move = true;
  cell.diffuse = false;

  auto mating = cell.addProperty<int>(-1); // -1 = P-type, 1 = M-type
  auto receptor_total = cell.addProperty<int>(600);
  auto receptor_occupied = cell.addProperty<int>(0);
  auto phase = cell.addProperty<int>(1); // 1 : G1, 2: S/G2, 3 = M
                                         // auto ADD_PROPERTY
  auto C = cell.addProperty<int>(0);
  auto Y = cell.addProperty<int>(0);
  auto Yp = cell.addProperty<int>(0);
  auto D = cell.addProperty<int>(Dtot_int - 1);
  auto Mi = cell.addProperty<int>(0);
  auto Ma = cell.addProperty<int>(1);
  auto Mr = cell.addProperty<int>(0);
  auto dtot = cell.addProperty<int>(1000);
  auto switching = cell.addProperty<int>(1); // 0 = don't switch, 1 = switch

/* setting size of the system */
  setSize(box_size);

  Vec up;
  up.at(0) = 0;
  up.at(1) = 0;
  up.at(2) = -1;
/* add a force, which holds the cells on a plane */
  add_force(F_FUNC(1E-23 * (box_size / 2 - A.position.at(2))),
    B_FUNC(A.type == cell.type));
/* add a force which allows pheromones only to diffuse above the plane */
  add_force(
    F_FUNC(1E-21 * up),
    B_FUNC((A.type == F_M.type || A.type == F_P.type || A.type == X.type) &&
    A.position[1] > box_size / 2));

/* creates a second cell once at the beginning of the simulation */
  reaction set_second_cell(as_A(cell));
```

```
  set_second_cell.rate = 100000 / units::second;
  set_second_cell.divide(as_B(cell), 1.E-8);
  set_second_cell.reacPossibleFunc = B_FUNC(
    nr_in_system(cell) == 1 && usedSystem->time < (100 * units::second));
  set_second_cell.afterFuncA = G_FUNC(mating(B) = 1; B.colortype = -2;);

/* cell cycle reaction (1-9) */
// 1) cyclin synthesis in cell   0 -> Y
  reaction Y_synthesis(as_A(cell));
  Y_synthesis.rateFunc = R_FUNC(k1);
  Y_synthesis.afterFuncA = G_FUNC(Y(A) = Y(A) + 1;);

// 2) formation of inctive MPF complex   D + Y -> M_I
  reaction form_MPF(as_A(cell));
  form_MPF.reacPossibleFunc = B_FUNC(Y(A) > 0);
  form_MPF.rateFunc = R_FUNC(k2 * Y(A) * D(A) / dtot(A));
  form_MPF.afterFuncA =
    G_FUNC(Y(A) = Y(A) - 1; D(A) = D(A) - 1; Mi(A) = Mi(A) + 1;);

// 3) activation of MPF complex   M_I + M_A -> 2 M_A
  reaction activation_1(as_A(cell));
  activation_1.reacPossibleFunc = B_FUNC(Mi(A) > 0 && Ma(A) > 0);
  activation_1.rateFunc =
    R_FUNC(k3prime * Mi(A) + k3 * Ma(A) / dtot(A) * Ma(A) / dtot(A) * Mi(A));
  activation_1.afterFuncA = G_FUNC(Mi(A) = Mi(A) - 1; Ma(A) = Ma(A) + 1;);

// 4) breakage of activated MPF complex M_A -> Y_p + D
  reaction breakage(as_A(cell));
  breakage.reacPossibleFunc = B_FUNC(Ma(A) > 1);
  breakage.rateFunc = R_FUNC(k4 / (volume(A) / V_0) * Ma(A));
  breakage.afterFuncA =
    G_FUNC(Ma(A) = Ma(A) - 1; Yp(A) = Yp(A) + 1; D(A) = D(A) + 1;);

// 5) cyclin degradation   Y_p -> 0
  reaction degradation(as_A(cell));
  degradation.reacPossibleFunc = B_FUNC(Yp(A) > 0);
  degradation.rateFunc = R_FUNC(k5 * Yp(A));
  degradation.afterFuncA = G_FUNC(Yp(A) = Yp(A) - 1;);

// 6) cell growth
  reaction growth(as_A(cell));
  growth.rateFunc = R_FUNC(k6);
  growth.afterFuncA = G_FUNC(volume(A) = volume(A) + V_0 / Td;);

// 7) cell cycle transition from G1->S/G2
  reaction phase_c1(as_A(cell));
  phase_c1.reacPossibleFunc = B_FUNC(Mi(A) > t7 && phase(A) == 1); //#M_I > t7
  phase_c1.rate = k7;
  phase_c1.afterFuncA = G_FUNC(phase(A) = 2;);

// 8) cell cycle transition from S/G2->M
  reaction phase_c2(as_A(cell));
  phase_c2.reacPossibleFunc = B_FUNC(Ma(A) > t8 && phase(A) == 2); //#M_A > t8
  phase_c2.rate = k8;
```

```
  phase_c2.afterFuncA = G_FUNC(phase(A) = 3;);

// 9) cell division (transition from M->G1)
  reaction division(as_A(cell));
  division.reacPossibleFunc = B_FUNC(Ma(A) < t9 && phase(A) == 3); // #M_A < t9
  division.rate = k9;
  division.divide(as_B(cell), 1.E-8);
  division.afterFuncA = G_FUNC(
    volume(B) = 0.5 * volume(A); phase(B) = 1; D(B) = D(A) / 2;
    Y(B) = Y(A) / 2; Yp(B) = Yp(A) / 2; Ma(B) = Ma(A) / 2; Mi(B) = Mi(A) / 2;
    Mr(B) = Mr(A) / 2; dtot(B) = D(B) + Ma(B) + Mi(B) + Mr(B);
    receptor_occupied(B) = receptor_occupied(A) / 2;
    volume(A) = 0.5 * volume(A); phase(A) = 1; D(A) = D(A) - D(B);
    Y(A) = Y(A) - Y(B); Yp(A) = Yp(A) - Yp(B); Ma(A) = Ma(A) - Ma(B);
    Mi(A) = Mi(A) - Mi(B); Mr(A) = Mr(A) - Mr(B); dtot(A) = dtot(A) - dtot(B);
    receptor_occupied(A) = receptor_occupied(A) - receptor_occupied(B););
  division.afterFuncB = G_FUNC(if (switching(A) == 0) {
    mating(B) = mating(A);
    switching(B) = 1;
    colorType(B) = colorType(A);
  } else {
    mating(B) = -mating(A);
    switching(B) = 0;
    colorType(B) = -colorType(A);
  });

// adds cdc2 to the cell, if it is growing
  reaction D_synthesis(as_A(cell));
  D_synthesis.reacPossibleFunc = B_FUNC(dtot(A) / volume(A) < Dtot_int / V_0);
  D_synthesis.rate = 200 / units::minute;
  D_synthesis.afterFuncA = G_FUNC(D(A) = D(A) + 1; dtot(A) = dtot(A) + 1;);

/* cell -> 0 */
  reaction death(as_A(cell));
  death.reacPossibleFunc = B_FUNC(nr_in_system(cell) > 1);
  death.rate = 0.003 / units::minute;
  death.Adead();

/* cell -> cell + F_M */
  reaction produce_F_M(as_A(cell));
  produce_F_M.rate = k10;
  produce_F_M.reacPossibleFunc = B_FUNC(mating(A) == -1);
  produce_F_M.createAt_A(F_M);

/* cell -> cell + F_P */
  reaction produce_F_P(as_A(cell));
  produce_F_P.rate = k10;
  produce_F_P.reacPossibleFunc = B_FUNC(mating(A) == 1);
  produce_F_P.createAt_A(F_P);

/* cell -> cell + X */
  reaction produce_X(as_A(cell));
  produce_X.rate = k10 / 10;
  produce_X.reacPossibleFunc = B_FUNC(mating(A) == -1);
```

```
  produce_X.createAt_A(X);

/* cell(n) + F_M -> cell(n+1) */
  reaction bind_pherome_M(as_A(cell), as_B(F_M));
  bind_pherome_M.barrier = 0.;
  bind_pherome_M.setOverlapp(0.01);
  bind_pherome_M.reacPossibleFunc =
    B_FUNC(receptor_occupied(A) < receptor_total(A) && mating(A) == 1);
  bind_pherome_M.afterFuncA =
    G_FUNC(receptor_occupied(A) = receptor_occupied(A) + 1;);
  bind_pherome_M.Bdead();

/* cell(n) + F_P -> cell(n+1) */
  reaction bind_pherome_P(as_A(cell), as_B(F_P));
  bind_pherome_P.barrier = 0.;
  bind_pherome_P.setOverlapp(0.01);
  bind_pherome_P.reacPossibleFunc =
    B_FUNC(receptor_occupied(A) < receptor_total(A) && mating(A) == -1);
  bind_pherome_P.afterFuncA =
    G_FUNC(receptor_occupied(A) = receptor_occupied(A) + 1;);
  bind_pherome_P.Bdead();

/* cell(n) -> cell(n-1) + F_M */
  reaction unbind_pheromone_M(as_A(cell));
  unbind_pheromone_M.rateFunc =
    R_FUNC(2 * 0.02 / units::minute * receptor_occupied(A));
  unbind_pheromone_M.divide(as_B(F_M), 1e-11);
  unbind_pheromone_M.reacPossibleFunc =
    B_FUNC(receptor_occupied(A) > 0 && mating(A) == 1);
  unbind_pheromone_M.afterFuncA =
    G_FUNC(receptor_occupied(A) = receptor_occupied(A) - 1;);

/* cell(n) -> cell(n-1) + F_P */
  reaction unbind_pheromone_P(as_A(cell));
  unbind_pheromone_P.rateFunc =
    R_FUNC(2 * 0.02 / units::minute * receptor_occupied(A));
  unbind_pheromone_P.divide(as_B(F_P), 1e-11);
  unbind_pheromone_P.reacPossibleFunc =
    B_FUNC(receptor_occupied(A) > 0 && mating(A) == -1);
  unbind_pheromone_P.afterFuncA =
    G_FUNC(receptor_occupied(A) = receptor_occupied(A) - 1;);

/* cell(Mi) -> cell(Mr) */
  reaction response(as_A(cell));
  response.reacPossibleFunc = B_FUNC(receptor_occupied(A) > 0 && Mi(A) > 0);
  response.rateFunc =
    R_FUNC(1.5 / units::minute * receptor_occupied(A) * receptor_occupied(A) *
    receptor_occupied(A) /
    (300 * 300 * 300 + receptor_occupied(A) * receptor_occupied(A) *
    receptor_occupied(A)) *
    Mi(A) * V_0 * V_0 / (volume(A) * volume(A)));
  response.afterFuncA = G_FUNC(Mi(A) = Mi(A) - 1; Mr(A) = Mr(A) + 1;);

/* cell(Mr) -> cell(Mi) */
```

```
    reaction response_back(as_A(cell));
    response_back.reacPossibleFunc = B_FUNC(Mr(A) > 0);
    response_back.rateFunc = R_FUNC(0.02 / units::minute * Mr(A));
    response_back.afterFuncA = G_FUNC(Mr(A) = Mr(A) - 1; Mi(A) = Mi(A) + 1;);

/* F_P -> 0 */
    reaction degradation_P(as_A(F_P));
    degradation_P.rate = k13;
    degradation_P.Adead();

/* F_M -> 0 */
    reaction degradation_M(as_A(F_M));
    degradation_M.rate = k13;
    degradation_M.Adead();

/* X + F_P -> X */
    reaction degradate_PX(as_A(X), as_B(F_P));
    degradate_PX.barrier = 0.;
    degradate_PX.setOverlapp(0.01);
    degradate_PX.Bdead();

/* x -> 0 */
    reaction degradation_X(as_A(X));
    degradation_X.rate = k13;
    degradation_X.Adead();

    Vec begin;
    begin.at(0) = box_size / 2;
    begin.at(1) = box_size / 2;
    begin.at(2) = box_size / 2;

/* diffusion out of the system for F_P, F_M and X */
/* F_P -> 0 */
    reaction diff_out_F_P(as_A(F_P));
    diff_out_F_P.rate = 0.001;
    diff_out_F_P.reacPossibleFunc = B_FUNC(abs(A.position - begin) > 24000);
    diff_out_F_P.Adead();

/* F_M -> 0 */
    reaction diff_out_F_M(as_A(F_M));
    diff_out_F_M.rate = 0.001;
    diff_out_F_M.reacPossibleFunc = B_FUNC(abs(A.position - begin) > 24000);
    diff_out_F_M.Adead();

/* X -> 0 */
    reaction diff_out_X(as_A(X));
    diff_out_X.rate = 0.001;
    diff_out_X.reacPossibleFunc = B_FUNC(abs(A.position - begin) > 24000);
    diff_out_X.Adead();

/* Adding objects to system */
    auto dummy_1 = putHere(dummy, 0, 0, 0);
    auto box_1 = putHere(Box, box_size / 2, box_size / 2, box_size / 2);
    putInsideAt(box_1, cell, box_size / 2, box_size / 2, box_size / 2);
```

```
/* Adding observations */
  observe::nr_of(F_P);
  observe::nr_of(F_M);
  observe::nr_of(X);
  observe::nr_of(cell, B_FUNC(mating(A) == 1));
  observe::nr_of(cell, B_FUNC(mating(A) == -1));

/* run the visualization */
  visualize(25);
  }
}
```

2.3 Lipid raft model

Listing 9: Model to simulate accumulation of proteins in a lipid raft with increased viscosity (lipidraft_model.cpp).

```
// 2D model
#include "simulator/dsl.h"
using namespace dsl;
namespace model {

void lipidraft_model() {
  set_temperature(300 * units::kelvin);
  set_viscocity(1E-3 * units::pascal * units::second);

  ADD_SPECIES(LRP5); // LRP 5/6
  radius(LRP5) = 3.7 * units::nano_meter;
  LRP5.local_density = nature::protein_density;
  colorType(LRP5) = 2;

  ADD_SPECIES(LR); // Lipid Rafts
  radius(LR) = 200 * units::nano_meter;
  LR.local_density = nature::protein_density;
  LR.local_viscosity = 10E-3 * units::pascal * units::second;
  colorType(LR) = 5;

  ADD_SPECIES(CK1); // CK1-gamma
  radius(CK1) = 2.4 * units::nano_meter;
  CK1.local_density = nature::protein_density;
  colorType(CK1) = 8;

  const double box_size = 709;
  setSize(box_size);

/* LR[] + CK1 -> LR[CK1] */
  reaction CK1_in(as_A(CK1), as_B(LR));
  CK1_in.barrier = 0. * units::kilogram * units::nano_meter *
    units::nano_meter / units::second / units::second;
  CK1_in.AinB();
```

```
/* LR[] + LRP5 -> LR[LRP5] */
  reaction LRP5_in(as_A(LRP5), as_B(LR));
  LRP5_in.barrier = 12.E-3 * units::kilogram * units::nano_meter *
    units::nano_meter / units::second / units::second;
  LRP5_in.AinB();

/* LR[CK1] -> LR[] + CK1 */
  reaction CK1_out(as_A(CK1), as_B(LR));
  CK1_out.barrier = 0. * units::kilogram * units::nano_meter *
    units::nano_meter / units::second / units::second;
  CK1_out.AleaveB();

/* LR[LRP] -> LR[] + LRP5 */
  reaction LRP5_out(as_A(LRP5), as_B(LR));
  LRP5_out.barrier = 0. * units::kilogram * units::nano_meter *
    units::nano_meter / units::second / units::second;
  LRP5_out.AleaveB();

  auto LR_test = putHere(LR, box_size / 2, box_size / 2, box_size / 2);
  putSomewhere(LRP5, 200);
  putSomewhere(CK1, 200);

/* Adding observations */
  observe::nr_of(LRP5);
  observe::nr_of(CK1);
  observe::nr_of(LRP5, B_FUNC(A.parentId != -1));
  observe::nr_of(CK1, B_FUNC(A.parentId != -1));

/* run the visualization */
  visualize(25);
  }
}
```

References

[1] Frank C. Collins and George E. Kimball. Diffusion-controlled reaction rates. *Journal of Colloid Science*, 4(4):425–437, August 1949.

[2] K. Razi Naqvi. Diffusion-controlled reactions in two-dimensional fluids: discussion of measurements of lateral diffusion of lipids in biological membranes. *Chemical Physics Letters*, 28(2):280–284, September 1974.

[3] Marian von Smoluchowski. Versuch einer mathematischen Theorie der Koagulationskinetik kolloider Lösungen. *Zeitschrift für physikalische Chemie*, (92):129 – 168, 1917.

[4] Osman N. Yogurtcu and Margaret E. Johnson. Theory of bi-molecular association dynamics in 2d for accurate model and experimental parameterization of binding rates. *The Journal of Chemical Physics*, 143(8):084117, August 2015.
